# Basic Research on Tendon Repair: Strategies, Evaluation, and Development

**DOI:** 10.3389/fmed.2021.664909

**Published:** 2021-07-28

**Authors:** Zhi Jie Li, Qian Qian Yang, You Lang Zhou

**Affiliations:** ^1^Research for Frontier Medicine and Hand Surgery Research Center, The Nanomedicine Research Laboratory, Research Center of Clinical Medicine, Department of Hand Surgery, Affiliated Hospital of Nantong University, Nantong, China; ^2^Medical School of Nantong University, Nantong, China

**Keywords:** tissue engineering, platelet-rich plasma therapy, growth factor and drug therapy, stem cell therapy, gene therapy, tendon healing, tendon injury, tendon

## Abstract

Tendon is a fibro-elastic structure that links muscle and bone. Tendon injury can be divided into two types, chronic and acute. Each type of injury or degeneration can cause substantial pain and the loss of tendon function. The natural healing process of tendon injury is complex. According to the anatomical position of tendon tissue, the clinical results are different. The wound healing process includes three overlapping stages: wound healing, proliferation and tissue remodeling. Besides, the healing tendon also faces a high re-tear rate. Faced with the above difficulties, management of tendon injuries remains a clinical problem and needs to be solved urgently. In recent years, there are many new directions and advances in tendon healing. This review introduces tendon injury and sums up the development of tendon healing in recent years, including gene therapy, stem cell therapy, Platelet-rich plasma (PRP) therapy, growth factor and drug therapy and tissue engineering. Although most of these therapies have not yet developed to mature clinical application stage, with the repeated verification by researchers and continuous optimization of curative effect, that day will not be too far away.

## Introduction

As an anatomical structure connecting muscle and bone, tendons are highly resistant to mechanical loads, and capable of transferring, distributing and regulating the forces exerted by muscles to the connected structures. In this way, tendons maintain posture or generate movement (1–3). Tendons, which connect muscles to bones, have high tear resistance and tensile strength and play a crucial role in the stable movement of bones. But in fact, tendons composed of cells and parallel arrays of collagen fibers are often injured and even ruptured (2). The total incidence rate of tendon or ligament injuries is about 1/1,000 per year. Up to 46% of musculoskeletal injuries are reported as tendon injuries, including tendinopathy (4). Due to the lack of sufficient cells and growth factors, tendon healing is slow and the quality is unsatisfactory (5, 6).

The arrangement of tendons is hierarchical. The fibers composed of triple helix type I collagen molecules form fibers, bundles and tendon units in turn. Type I collagen is the most abundant ingredient of the extracellular matrix (ECM) in all soft tissues, including tendons. The special framework of the tendon is counted on the specific parallel organization of type I collagen fibrils, instead of the expression of type I collagen. So far, little is known about the mechanism by which type I collagen fibers drive specific spatial structures in tendons. Growth factors, such as transforming growth factors (TGF)-β and fibroblast growth factors (FGFs), are beneficial to improve the expression of collagen and promote the synthesis of collagen in tendon tissue. The synthesis of type I collagen in tendons also involves mechanical forces: increased load leads to increased collagen content in the tendons and reduced load results in reduced collagen content (1).

Collagen fibers densely arranged in ECM are considered as the primary force transfer unit of the tendon (1). A variety of ECM molecules are involved in the formation of type I collagen, including collagen, elastin and glycoprotein (7). Human tendon tears at 8% strain and plastic deformation occurs at 4% strain (8).

Based on previous surveys, tendon injuries are considered as the most common musculoskeletal disorder for patients seeking medical treatment (9, 10). For example, 1 of every ten people and 1 of every two runners are afflicted with Achilles tendinopathy before the age of 45 (11). Tendon injuries can be caused by sudden rupture orchronic process, which is widely known as tendinopathy (2, 3).

Acute mechanical load tends to result in the outbreak of tendon tears (2, 3). In contrast, the pathogenesis of tendinopathy or chronic tendon injury is still controversial and has different definitions, degenerative disease or failure of the healing process (12–14). Chronic tendon injury or tendinopathy refers to the clinical symptoms of affected tendons, including pain, focal tendon tenderness, decreased strength and reduced activity. There is no macroscopic tear at the time of tendinopathy in comparison with partial or complete tendon tears. The following histological features can characterize tendinopathy: disordered collagen fibers, increase of proteoglycan and glycosaminoglycan content, increase of non-collagenous ECM, cell proliferation and neovascularization (15–18).

Both chronic and acute tendon injuries have many extrinsic and intrinsic factors (14). Common inherent risk factors for tendon diseases include sexual distinction, age, type 2 diabetes and obesity (14, 19). The main extrinsic factor of tendon injury is abnormal tendon load related to physiological exercise and specific working environment. Tendinopathy is thought to be caused by repeated eccentric mechanical loading, while acute tendon injury usually occurs after a single overloading event (2, 3, 13, 19–22).

Tendon injuries could bring a high burden to patients leading to a significant loss in individual production capacity. Moreover, the quality of patients' life is impaired, and the whole society's healthcare system suffers huge losses (23).

The natural healing process of injured tendons is complex and varies in clinical outcomes according to the anatomical location of the tendon tissue. The wound healing process comprises three overlapping phases: the wound healing process, proliferative phase, and tissue remodeling phase (24). In the inflammatory stage, red blood cells infiltrate into the wounded area, accompanied by white blood cells (leukocytes), and platelets equipped with important growth factors. Tendon cells, also known as tenocytes, recruited to the wound site are stimulated to multiply, especially in the epitenon (24). Inflammatory cells secrete a variety of cytokines to promote tendon healing, stimulate cell migration and angiogenesis. These cytokines include insulin-like growth factor-1 (IGF-1), transforming growth factor-beta (TGF-β), platelet-derived growth factor (PDGF) and basic fibroblast growth factor (bFGF) (25–27). The second stage, known as the proliferative or repair stage, is characterized by a large amount of synthetic activity under the guidance of macrophages and tenocytes. Macrophages that alter from phagocytic to reparative release growth factors (16, 28). The synthesis of collagen I begins to play a leading role in the third and final stage known as the remodeling phase. This phase starts 1–2 months after injury and spans more than 1 year. The repaired tissue is similar to a scar in appearance. The biomechanical properties of the repaired tissue can never restore entirely because of an increase in the water content and a decrease in the quantity and quality of collagen (16). As a result, rupture may occur later, and sometimes a reduction in load capacity is observed, which is due to the tendency of tendons to form adhesions (29, 30). The healing process of injured tendons is often longer and the healing intensity is weak in the early stage owing to the lack of cells and the low activity of growth factors (5, 31). In addition, the mechanical properties of the healed tendon were only 70% of that of the pre-injured tendon (32, 33).

The development of new treatments is imminent. In this review, we introduce various therapies' research progress and achievements in recent years from various aspects.

## Treatment of Tendon Injuries

The first-line treatment options are different facing acute and chronic tendon injuries. The main purpose of chronic tendon injury treatment is to reduce pain, mainly through local or systemic anti-inflammatory drugs, while treatment of acute tendon injuries aims to repair broken tendons with surgical techniques (3, 34, 35).

In terms of tendon treatment, the management of minor injuries may be relatively straightforward and a combination of moderate rest and/or medical intervention is usually enough. Surgery is considered the last resort for tendon disease. The type and location of the injury determine the outcome of reconstruction surgery (3). There are many suture techniques including four-strand cross-lock repair, *U*-shaped repair, four-strand Kessler type repair and etc. The four-strand cross-lock and *U*-shaped repair methods may be better (36). Even though, more than 40% of surgical patients still have complications and limited functionality after tendon surgery (37–40).

Faced with such situation, it is urgently needed to find out advanced therapies. Several methods have been investigated to enhance the healing process including drugs, growth factors, gene therapy, stem cell therapy, platelet-rich plasma (PRP) therapy, and tissue engineering.

### Drugs

In tendon tears, the ruptured tendons generally do not require medical treatment. But it seems to be about to change. In 2012, Bryan et al. found that within 6 weeks, compared with conventional sutures, the tendon repaired with Butyric Acid (BA)-impregnant suture showed better biomechanical properties (41). Although the U.S. Food and drug administration has not yet approved clinical use, BA is a natural carboxylic acid that has been proved to enhance angiogenesis in appropriate concentrations (42). In the early biological healing of tendon repair, the BA-impregnant suture can improve the biomechanical strength of repaired tendon, so as to play a protective role in early rehabilitation. Curcumin contributes to complete tendon healing not only histologically but also biomechanically. That is the reason why it may be an additional drug for the surgical repair of tendon injury (43).

In chronic tendinopathy, the main purpose of drug therapy is to relieve pain, such as NSAIDs (Non-steroidal Anti-inflammatory Drugs), but it does not alter long-term outcomes. However, as one of the drugs, glucocorticoid injection has been proved to have significant long-term damage to tendon tissue and cells. A 2014 study showed that the local application of glucocorticoid had obvious adverse effects on tendon cells *in vitro*, including decreased cell viability, cell multiplication as well as collagen synthesis (44).

Besides, there are also some potential discoveries in recent years. A study in 2018 showed that tendons could acutely alter glucose metabolism, increasing glycolysis and lactic acid synthesis to deal with injury. This study also demonstrated that lactic acid synthesis inhibitors could promote the recovery of biomechanical properties of injured tendons (45). In 2017, Tack et al. demonstrated the role of direct (synthesis rate modification) and indirect (antioxidant) mechanisms in tendon healing in animal models by studying the effects of amino acids and vitamins, effects of amino acids and vitamins (46).

### Growth Factors

As mentioned above, growth factors can be used independently or be involved in other kinds of therapies (47). These growth factors control tendon healing with the help of many regulators (2, 48). So far, there is a lot of knowledge about growth factors, such as TGF-β, bFGF, VEGF (vascular endothelial growth factor), and PDGF, through various studies both *in vivo* and *in vitro* (49).

There are three major isoforms of TGF—β, which is involved in a variety of cellular pathways (49). It is well-known that TGF—β is engaged in a series of reactions, including initial inflammatory response, collagen synthesis, neovascularization and fibrosis/excessive scar formation (49). TGF—β1 is one of three isoforms expressed by tenocytes, inflammatory cells and infiltrating fibroblasts (50, 51). According to the present understanding, TGF—β1 is closely related to the occurrence and development of excessive scar formation. It is worth mentioning that after flexor tendon injury, when TGF—β1 signaling pathway is blocked by antibody or miRNA, the range of finger movement is improved, but the mechanical strength of tendon is downregulated (50, 52, 53). Type 2 and type 3 of transforming growth factor are vital for tendon formation and have the potential to induce tendon progenitor cells (54, 55).

Basic fibroblast growth factor (bFGF), also known as FGF2, is a member of heparin-binding growth factor family. As a single chain polypeptide, bFGF promotes a variety of mitotic and angiogenic activities (5, 56). FGF2 is related to inflammation, cell proliferation, angiogenesis and collagen synthesis during tendon healing (57–62). The role of exogenous FGF in tendon injury remains controversial. Ectopic FGF2 is considered to be able to promote cell proliferation and angiogenesis during tendon repair, but the improvement of mechanical strength is still unclear (62, 63). However, some studies indicating that exogenous administration of FGF2 through a fibrin-heparin-based delivery system cannot improve the mechanical or functional properties of flexor tendon injury in dogs (60).

The VEGF family comprises several isoforms that linked to three tyrosine kinase receptors and their isoforms determine the bioavailability for each receptor (64). During the process of tendon development, VEGF levels ascend. The presence of VEGF in human fetal tendon is considered to be the cause of the differentiation of vascular and non-vascular regions in the tendon (65). Then VEGF levels descended to a steady state in the adult Achilles tendon (66). The lowest increase level of VEGF in adults means a long-term overuse tendon injury (67). It has been confirmed for a long time that the levels of VEGF increase early during tendon healing. VEGF promotes neovascularization by stimulating matrix metalloproteinases, which may degrade connective tissue and enhance angiogenesis (66, 68–70). Ectopic VEGF delivery can improve the strength of tension injured Achilles tendon. However, VEGF did not significantly up-regulate the expression of the collagen gene. Therefore, it is not necessarily the most critical factor in the synthesis of collagen during intrasynovial tendon healing, it just plays a vital role in angiogenesis during the repair and regeneration of injured tendons.

As a 30-kDa dimer, PDGF contributes to the migration and proliferation of fibroblasts, tenocytes and mesenchymal stem cells related to tissue homeostasis (71). PDGF signaling pathway may be essential to tendon homeostasis. It has been proved that downregulation of PDGF signal inhibited the normal growth response of tendon tissue to mechanical stimulation in adult mice (72). The role of PDGF in tendon development is not well-understood. In many animal and tendon models, exogenous delivery of PDGF contributes to the morphological and biomechanical properties, indicating that PDGF may help to enhance tendon healing (73–77).

In addition to the above four, many growth factors also play a role in tendon healing, such as CTGF, IGF and EGF (49). Moreover, multiple factors can be applied simultaneously to achieve better results. Recently, growth factors are more likely to be used in conjunction with gene therapy, cell therapy and tissue engineering than in isolation.

### Gene Therapy

Despite recent medical advances, tendon tissue repair and regeneration remained a relatively unattainable challenge (78). In the past few decades, many surgeons and researchers have tried some methods to accelerate tendon healing and prevent tendon adhesion, such as biodegradable synthetic biomaterial barrier, inhibitors and gene therapy (79–83). Among these therapies, gene therapy is considered as a promising method (84).

Gene therapy is the treatment of disease by introducing a foreign nucleic acid (such as DNA or RNA) into a specific cell/tissue. Considering their negative charge and considerable size, these molecules require gene vectors to mediate their transfer (85).

The basic principle of gene therapy in tendon healing is that when a tendon is damaged, genes critical for collagen production cannot be activated in time. In addition, genes that cause excessive scarring during tendon healing may be overexpressed after injury, leading to an unbalanced tendon healing process. For more than a decade, the approach behind gene therapy has been to correct this balance to achieve early healing strength with the lowest possible adhesion around the tendon after direct end-to-end surgical repair (86).

Founded on the concept of offering therapeutic gene sequences, gene transfer procedures can be used to promote tendon healing, because these gene sequences can persistently increase the healing response and restore the tendon function before injury as completely as possible. Due to a deeper understanding of tendon biology, physiology, pathophysiology and tendon repair mechanism, there has been researches for the advantages of gene therapy (87).

The advantage of gene therapy is that the continuous expression of genes can increase the endogenous gene products in target tissues, such as signal molecules and transcription factors (3). Compared with stem cell therapy, gene therapy has a lower incidence of immune responses (88). Gene therapy can produce continuous local production and secretion of proteins. This distinguishing feature allows the restriction of protein delivery associated with short half-lives to be bypassed and makes it possible to regulate the timing of bioactivity cues in tissue sites. Therefore, gene therapy is a promising approach for tendon regeneration (78).

**Figure 1:**
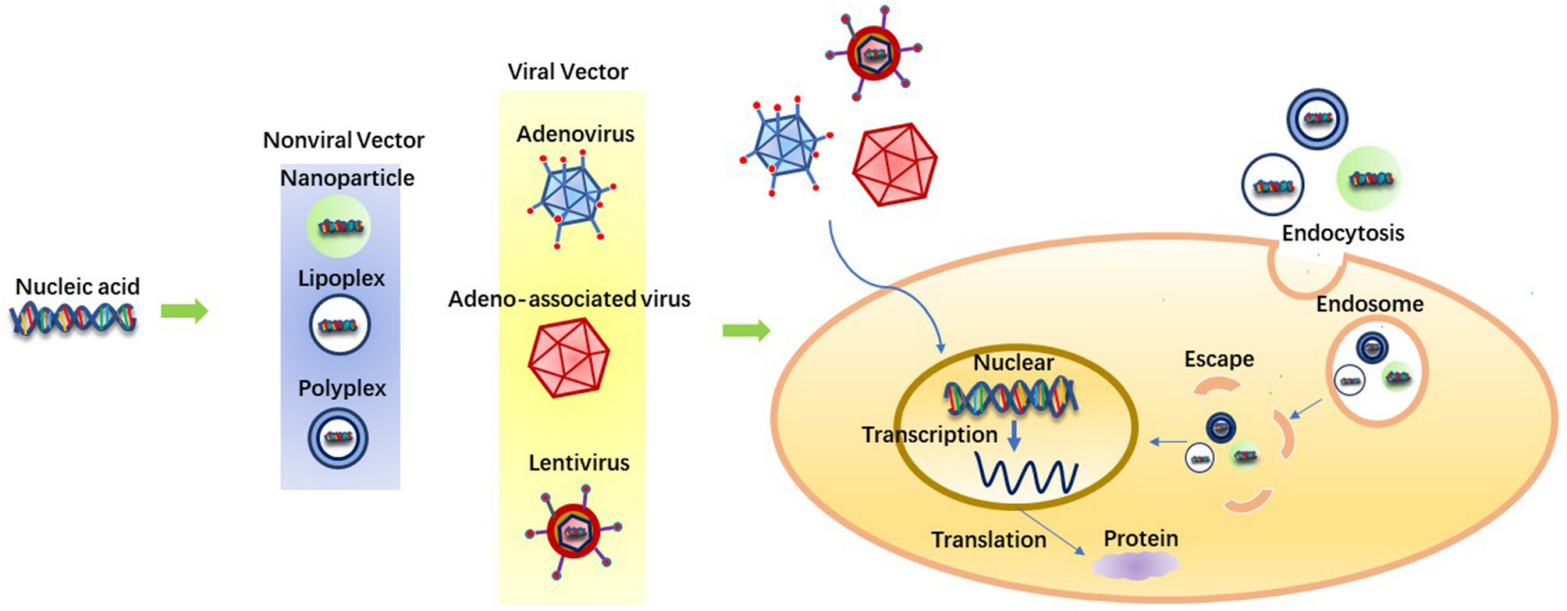
Different vectors in gene therapy and the procedure of gene delivery.

As is shown in Figure 1, there are three essential factors in gene therapy: target gene, gene carrier and target cell. Genes usually have a large molecular weight and a high negative charge density, so cell permeability is low, and gene therapy is limited (89, 90). In addition, the safe and effective delivery of nucleic acids into tendon tissues still needs to be considered. Basing on the consideration of biocompatibility and further improving the efficiency of gene therapy, a variety of vectors have been developed, including nanospheres, viral vectors and other delivery systems (91–94).

The selection of gene delivery vectors for tendon tissue repair and regeneration is an important parameter. Gene delivery vector dramatically affects the efficacy of gene therapy (95, 96). Many vectors have been tested for gene therapy, both viral and non-viral. Generally speaking, viral vectors are efficient in gene transduction, but there are safety hazards that are caused by virus insertion mutation and quality control (85, 97, 98). Although non-viral vectors are relatively safe, there is still an urgent requirement for them to improve transfection efficiency and expression of the transgene to be applied in clinical trials (99).

Many viral vectors such as adenovirus, adeno-associated virus (AAV), and lentivirus have been applied in animals to treat tendon injuries (100–102). As a typical viral vector, Adenovirus (Ad) can transduce many dividing and non-dividing cells, with solid but transient gene expression. Ad carrier is still the most familiar carrier in clinical trials (103, 104). In 2003, Ad became the basis for the first approved commercial gene therapy drug in China. Shenzhen GenTech has developed a modified Ad carrier Geneticine that encodes the p53 tumor suppressor gene and has been approved to treat head and neck cancer. Because of the lack of available information on clinical outcomes, the outcomes associated with this therapy remain controversial (105, 106). Although Ad is typical in clinical trials, it has several fundamental limitations. First, proteins in the viral capsid activate a strong immune response, and second, cell transduction is dependent on the presence of the CAR (coxsackie and adenovirus receptor) on the cell membrane, which leads to preferential hepatocyte transduction after intravenous injection. Finally, biological activity in the body is limited over time (107, 108).

Due to the side effects of viral vectors, non-viral vectors have been paid more and more attention because of their low immunogenicity, safety, flexibility of chemical design, stability and high gene capacity. In addition, non-viral vectors are easily created and chemically altered extensively. Finally and most importantly, the non-viral vector system is not limited by the size of the introduced gene molecule (85). Plasmid DNA (pDNA) is a circular, double-stranded DNA that replicates independently of chromosomal DNA. Based on the specific application, pDNA can be easily designed to express one or more genes. Compared with viral delivery systems, pDNA offers several advantages, including the ability of plasmids to hold significant genes (109). PLGA poly(lactic-co-glycolic acid) nanospheres have been shown to deliver plasmids efficiently to cultured muscle cells and injured tendons. Nanospheres ensured the high level of transgene expression in tendons for at least 6 weeks, and almost no tissue reaction was observed in tendons. It may be a promising non-viral vector for gene therapy (110). Nanospheres can achieve efficient gene-targeted delivery and protect gene sequences from degradation owing to its subcellular structure, which makes it easy to penetrate into the cell through endocytosis, enter the cytoplasmic chamber, and release the contained substances for long, so as to break through the absorption barrier of cell membrane (111–113). Cationic lipids and cationic polymers can also be used as non-viral vectors to carry DNA to form lipoplex and polyplex (114, 115). Many natural and synthetic polymers have been successfully used as delivery vectors. Since naturally occurring polymers are isolated from plant, animal or human tissues, vehicles usually developed from these polymers have the advantage of reproducing the critical structure and/or biochemical characteristics of ECM due to their natural derivation. Therefore, naturally occurring polymers have the advantages of low immunogenicity, relatively high abundance and easy processing. Fibrin and collagen are examples of such polymers (116, 117). Hydrogels and scaffolds can be formed in a mild manner to encapsulate more sensitive genetic vectors and cells. Synthetic polymers have the unique advantages of repeatability and controllable production, allowing precise operation (117–119). In addition, nuclear targeting of non-viral vectors can improve transfection efficiency by the application of SV40DNA-targeting sequences (DTS). DTS stimulates nuclear entry by binding nuclear localization signals to transcription factors, thereby encapsulating plasmid DNA and guiding nuclear entry (85).

Each vector has its inherent advantages and disadvantages, but the combination of biomaterial carriers can help to make up for the defects. Some studies have used surface coating and/or hydrogel encapsulation to mask Ad from host immune responses in order to compensate for the deficiency of viral vector Ad (78). Some researchers have tried to combine biomaterials such as fibrin and collagen with physical methods to treat musculoskeletal injury and have shown the potential of this approach (120–125).

Integration of biomaterial systems that appear in gene delivery strategies is central to most MS tissue engineering strategies and plays a role in the delivery system of both viral and non-viral vectors (78, 126). These biomaterials offer a combination of mechanical structure, cell support and biochemical signal control. In order to achieve this goal, biomaterial structures have been designed to be similar to natural extracellular matrix in structures (78).

As is shown in Table 1, gene delivery strategies have concentrated on sequences that are significant to tendon healing, such as growth factors, matrix molecules, transcription factors, anti-inflammatory molecules, and signaling molecules (145). It has been proved that bFGF and vascular endothelial growth factor A(VEGFA) are the most effective stimulants (146, 147). The transfer of bFGF and VEGFA genes can correct the deficiency of tendon intrinsic healing ability, and promote tendon healing, respectively (62, 94). In addition, it is worth mentioning that fibromodulin may be a good substitute for tendon healing growth factor (130).

**Table 1:** Research on gene therapy in recent years.

**Year**	**System**	**Gene**	**References**
2007	Adenovirus	BMP-14	(100)
2008	Adeno-Associated Virus-2	bFGF	(94)
2009	Bone marrow-derived mesenchymal stem cells (BMSCs) transduced with a type-five, first-generation adenovirus	TGF-β1, VEGF	(127)
2010	Mesoporous silica nanoparticles	PDGF	(77)
2014	Non-viral vectors	Scleraxis	(128)
2015	Non-viral vectors	CXCL13	(129)
2015	Plasmid complexed with histidylated liposomes.	Fibromodulin	(130)
2016	RV retroviruses/LV lentiviruses,	shRNA TOB1	(131)
2017	Plasmid	Tenomodulin	(132)
2018	PLGA nanoparticles	bFGF, VEGFA	(133)
2019	Gene-Loaded Nanoparticle-Coated Sutures	bFGF, VEGFA	(134)

*BMP, bone morphogenetic protein; bFGF, basic fibroblast growth factor; TGF-β1, transforming growth factor-beta 1; VEGF, vascular endothelial growth factor; PDGF, platelet derived growth factor; CXCL13, chemokine (C-X-C motif) ligand 13*.

In conclusion, gene therapy is an attractive strategy to promote tendon healing. The study of adaptive preclinical animal model has proved the feasibility of using this gene-based therapy in the treatment of tendon injury, which provides a reasonable hope for its transformation to patients as soon as possible.

### Stem Cell Therapy

In the management of chronic musculoskeletal diseases, regenerative medicine is a new modality which attracts increasing interest (148, 149). Stem cells have been widely used in the treatment of musculoskeletal diseases (150, 151). Current evidence suggests that stem cell therapy is highly effective on musculoskeletal disorders (152, 153). Stem cells are considered as cells that have the ability to divide and self-renew over a long period of time and capable of differentiating in all cell lines (154, 155). Stem cells have been assumed to promote regeneration during tendon healing (156, 157). The application of stem cell helps regulate inflammation, organize ECM regeneration, and promote tissue regeneration over scarring (158, 159). Identification of cell origin and characterization is necessary to achieve more effective repair or regeneration (160).

**Figure 2:**
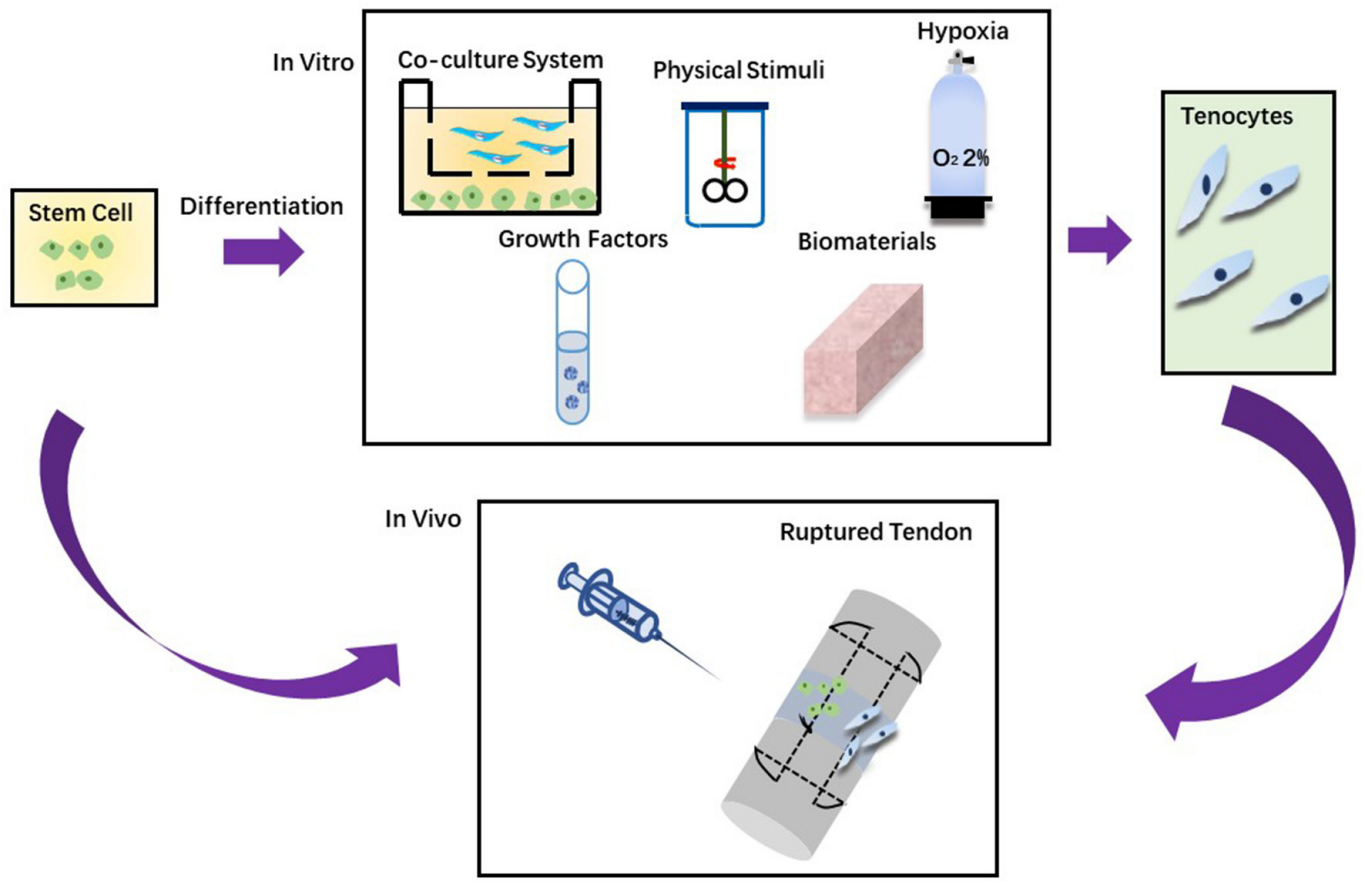
Two approaches in stem cell therapy. **(A)** Stem cells are injected directly into the injured tendon. **(B)** Stem cells are grown *in vitro* to differentiate into tenocytes and then injected into the injured tendon.

Stem cell-based therapies have attracted massive attention in the regeneration of defective tissues or organs. At present, stem cell-based regenerative treatment mainly includes isolation and screening, *in vitro* culture and amplification, and transplantation with or without directional differentiation, which is shown in Figure 2 (161–164). The success of gene therapy with relevance to applications for tendon healing can be improved with the role of the antiaging gene Sirtuin 1 that is critical for healing and regeneration. The activation of this genes is critical to the success of stem cell therapy (165–168). However cell transplantation has encountered many obstacles in therapeutic translation, including immuno-rejection, pathogen transmission, potential tumorigenesis, problems related to packaging, storage and transportation, and tissues in clinical application and regulatory approval (162, 169–173).

One of the fundamental problems limiting cell-based therapy is how to deliver cells to their targets and how they are implanted (174). Stem cells are either injected throughout the body into the circulation or injected locally at the injury site. Systemic administration of stem cells is limited by inefficient targeting of the injured area. At the same time, local injection may additionally do harm to the injured tissue. Because of implantation problems, survival rate of stem cells after local injection is low (174). With simple drug administration, tendon ruptures provide an attractive opportunity to target the site needed for treatment. Granulation tissue (i.e., the early loose connective tissue formed between the ends of the broken tendon) is composed of new capillaries (175). These newly formed capillaries have special molecular frameworks on surfaces, targeting ligands binding in an organ-specific manner (176). Therefore, in the proliferation phase, a random peptide library is filtered by phage display *in vivo* on the ruptured Achilles tendon and patellar tendon to look for the systemic administration peptide that is able to home to the injury site (176). At present, the best vascular peptide can home to damage tendons up to 200 times, and has been successfully used to deliver therapeutic agents that promote tissue regeneration (176–179). After these vascular targeting peptides were applied, the delivery and implantation of MSC to infarcted myocardium increased by four times (179). Therefore, a sizeable microvascular network resulted from a strong angiogenesis reaction at the rupture sites of tendons provides a large number of molecular targets for systemic administration of ligands, and transports the goods to the injured tendon (180). Neovascularization is a sign of tendinopathy Since these vascular targeting peptides can home to ruptured tendons, the platform for transporting stem cells to tendon injuries by using vascular homing peptide technology is basically already in place (174, 179).

There are many kinds of stem cells with different characteristics. Table 2 lists some of the categories. Some differences between stem cell populations must be noted.

**Table 2:** Research's on tendon stem cell therapy in recent years.

**Year**	**Stem cell**	**Strategy**	**References**
2015	MSC	Hypoxia	(135)
2016	TDSC/TSPC	Co-culture System	(136)
2018	ASC	Physical Stimuli	(137)
2018	TDSC/TSPC	Biomaterial	(138)
2019	MSC	Growth Factors	(139)

MSCs have recently been shown to possess more significant and possibly more crucial therapeutic functions in the injury response, such as immune modulation and nutritional activity. Therefore, they are defined as “drugstores” (181). In fact, they can enter the site of inflammation or tissue damage and begin to secrete immunomodulatory and nutritional substances, such as cytokines and growth factors, to rebuild physiological homeostasis to cope with the environment (181). Therefore, whether as a direct participant in the process or the “Drugstore” of bioactive molecules, BMSCs may enhance tissue repair and regeneration, thus restoring normal intra-articular homeostasis. These characteristics, together with the relatively easy isolation and amplification process, make MSCs have great potential in many clinical applications in recent years.

In 2003, 6.4 × 10^5^ of autologous bone marrow mesenchymal stem cells (BMSCs) were transplanted into SDFT of horses injured by strain for the first time (160). Isolated from bone marrow, BMSCs are applied and studied most commonly of all adult stem cells. BMSCs have been proven effective in regenerating different tissues, including tendons, either on their own or in combination with scaffolds (182, 183).

Adipose tissue-derived mesenchymal stem cells (ASCs) are the most abundant source of mesenchymal stem cells. Additionally, the higher number of mesenchymal stem cells extracted from the same amount of fat compared with bone marrow is another advantage of using ASCs. Because ASCs can produce and secrete components of ECM and cytokines, ASCs, as the most potential cells for cell therapy, have attracted much attention (184). In addition, compared with other mesenchymal stem cells, ASCs have the highest expression level of tendon ECM component and can be a hopeful cell source for the treatment of tendinopathy (185).

A recent study has shown that Tendon-derived MSCs (TDSCs) also known as tendon stem/progenitor cells (TSPCs) is an ideal cell type. Compared with other mesenchymal stem cells, TDSCs can display tendon-like phenotype and express the most tendon-related markers (186, 187). TSPCs are pluripotent adult stem cells involved in tendon healing (188, 189). It has been reported that TSPCs have clonality, differentiative potential and express specific surface markers of stemness (190). In addition, these TSPCs also express tenogenic markers making them a unique stem cell population. The number of TSPCs decreases with age, which may be the reason for the high prevalence of tendinopathies in the elderly (190, 191). *In vitro* and animal experiments, TSPCs are capable of differentiating in tenocytes (136, 192). TSPCs account for about 4% of the total number of tendon cells, (193). However, their use may be limited due to donor site lesions, an insufficient number of long-term cultured cells, and phenotypic drift during *in vitro* amplification, expansion is required before injection to *in vitro* (194).

The clinical potential of embryonic-like stem cell (ESC) to treat tendon injuries has been revealed, and the non-tumorigenic bias of these cells remains to be studied in more extended follow-up studies. Still, they require destruction of embryos to be isolated (195–197). Moreover, it has been demonstrated that they can differentiate into tendon cells (4).

Stem cells can be utilized to enhance the repair and regeneration of injured tendons. Different techniques can be applied to induce tendon differentiation and a gradual process can be avoidable to accidental differentiation. As shown in Table 2, there are many different strategies for tendon differentiation, such as hypoxia, biomaterials, growth factors, physical stimuli and co-culture system (198).

Despite many expectations, stem cell therapy has also been questioned. As is shown in a review in 2017, stem cell therapy still needs a lot of practice research before clinical application. Furthermore, there are also concerns about the long-term safety of stem cell therapy (199).

### Platelet-Rich Plasma (PRP)

The application of PRP is based on changing the molecular environment by providing a super-physiological concentration of platelets (and optional white blood cells) at the lesion/pathological site to simulate the initial stage of healing. The core of PRP research is composed of the molecular complexity of PRPs and their interactions with different forms of host tissue. PRP can manipulate and boost healing if we integrate the molecular knowledge of PRP and healing mechanism that PRP involved in,. Another layer of complexity, which is very challenging for researchers, describes the state of the host tissue: the stage of the disease and the mechanism of pathological tendon changes with which PRP is obliged to interact (200). There is such a probability that PRP composed of high concentrations of platelets, growth factors and cytokines, mainly by changing the molecular microenvironment, may contribute to healing.

One of the early effects of PRP on rats is intermittent inflammation. The activation of pro-inflammatory TNF alpha and NFκB pathways following PRP exposure, along with the expression of genes related to cell proliferation and tendon collagen remodeling have been observed (201). In chronic tendon diseases, the induction of acute inflammation may be a crucial factor in triggering the subsequent regeneration reaction (145). The inflammatory process seems to be under control of PRP from one of the key elements, hepatocyte growth factor (HGF). It is recognized that PRP has anti-inflammatory effect on human chondrocytes by inhibiting NFκB activation (202).

In the past two decades, PRPs have emerged in different medical fields as biotherapy for repairing or regenerating damaged or non-functional tissues. From the beginning, they have been used by highly expected sports medicine physicians and orthopedic surgeons to accelerate tissue healing (203). Anecdotal results from media coverage in elite athletes and other celebrities have increased the appeal of the public (204).

However, scientific understanding of how PRP works and clinical evidence to specific indications are not available yet and the process is time-consuming (200). A recent systematic review has underlined the controversial results of PRP applied in different pathologies. The authors assert that, in terms of current evidence, patellar and lateral elbow tendinopathy are benefited from PRP treatment, while in the treatment of Achilles tendon and rotator cuff, it seems that PRP do not help the conservative treatment or surgery (205). On the other hand, many studies have shown that PRP is conductive to Achilles tendon and rotator cuff disease (206, 207). Recently, a trial in the British Medical Journal (BMJ) disappoints PRP-related researchers reported that platelet-rich plasma did not provide any benefit after acute Achilles tendon rupture. PRP as an autologous whole-blood product composed of high concentrations of platelets, growth factors and cytokines is extensively used in sports medicine. The study included 230 adults from 19 British hospitals who suffered from acute Achilles tendon rupture in the previous 12 days without surgery. Patients were randomly given platelet-rich plasma injection or placebo and received standard rehabilitation care. At 24 weeks, there were no differences in tendon function, patient-reported function, pain, goal achievement, or quality of life between the two groups (208).

In summary, the future of PRP in tendon pathology remains open. At least, its role in the early stage of tendon healing has been proved by basic science studies. Still, a lot of preparation, for example, the preclinical premise and specific standardized clinical protocols is needed before it can be used in the clinical stage.

### Tissue Engineering

In 1997, Charles Vacanti Laboratory of the University of Massachusetts Medical Center created a mouse with the human ear on its back. The idea of manufacturing renewable tissue from *in vitro* has ushered in a new epoch in biomedical science, which is known as tissue engineering. As is first introduced in 1987, the concept of tissue engineering was defined to apply the principles and methods of engineering and life sciences to basically understand the structure-function relationship of normal and pathological tissues in mammals, and develop biological substitutes that can restore, keep or improve function (209).

Tissue engineering is a multidisciplinary approach aimed at inducing tissue repair, replacement, or regeneration. Tissue engineering involves using a combination of cells, scaffolds, and bioactive molecules to produce functional tissue (210).

The torn tissue will be replaced with tendons from other parts of the body in the early stage, tendons from another person, tendons from different species, or artificial tendons. As shown in Table 3, these grafts include autograft, allograft, xenograft and synthetic grafts (211). The main drawback of autografts is donor site complications. Allografts and xenografts are limited by the availability of donor tissues and the potential risk of immune rejection and pathogen transmission (3). A clinical trial has shown that the application of xenograft still needs to reduce its high infection rate (212). Early synthetic grafts have some disadvantages, such as early re-tear, decrease of mechanical strength with time, insufficient tissue that grows inward and graft debris deposition (213). Although the new generation of grafts shows better performance, the application of these artificial grafts remains controversial because clinical reports have not yet reached a unified and precise conclusion (214).

**Table 3:** Research's on tissue engineering treatment of tendon in recent years.

**Year**	**Graft**	**References**
2015	Autograft	(140)
2015	Xenograft	(141)
2015	Allograft	(142)
2018	Biomaterial	(143)
2020	Scaffold	(144)

**Figure 3:**
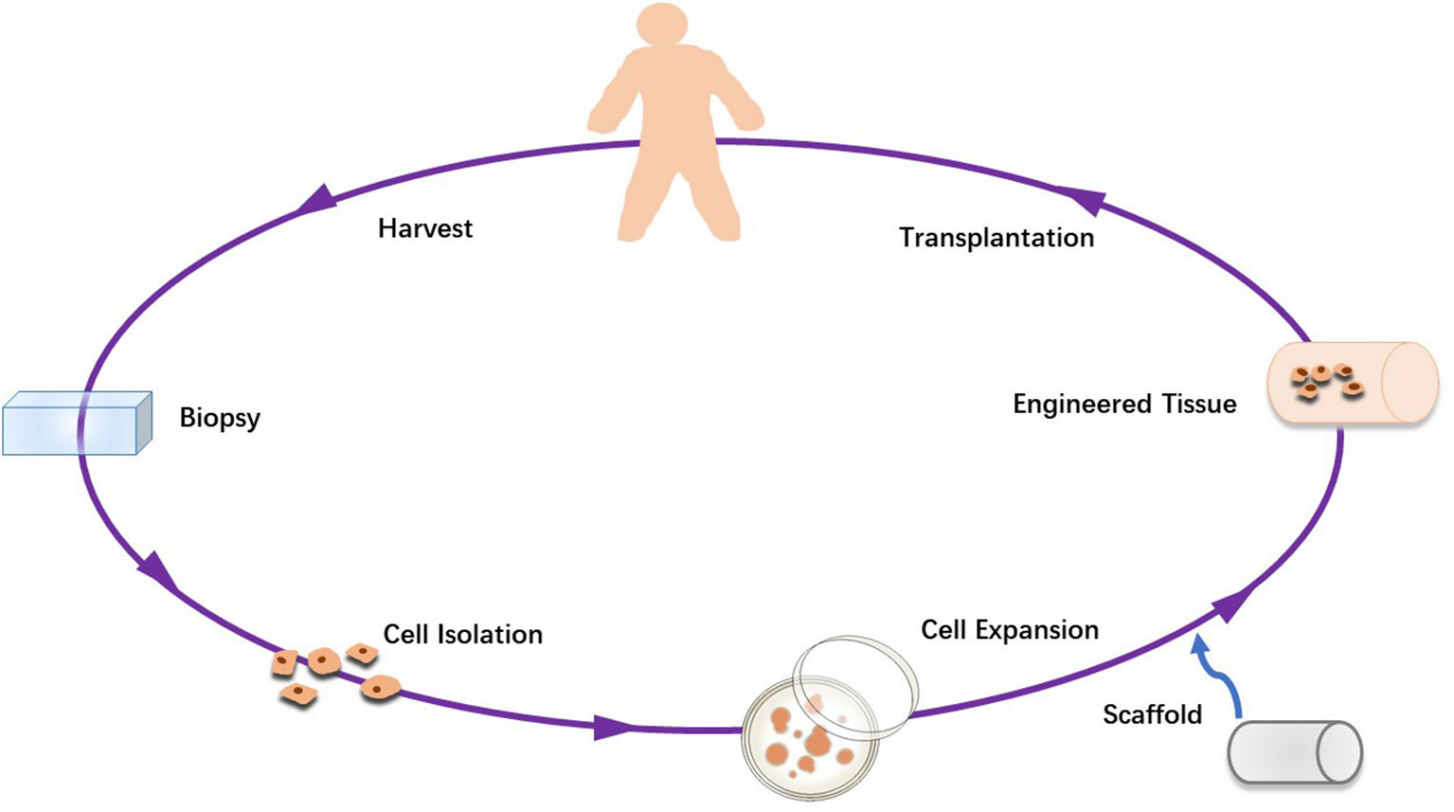
Schematic diagram of tissue engineering.

Because of the problems above, there has been increasing interest in the preparation of tissue-engineered tendons in the past decade. The concept of tissue engineering is to create a safe and effective substitute for tissue injured in the biological environment (215). Tissue-engineered grafts consist of two or all of the three main components: cells, biomaterials/scaffolds, and biomolecules (216). The fabrication of an engineered scaffold is a method of tissue-engineering grafts, which provides a physical environment to regulate the repair and regeneration of damaged tissue. Figure 3 shows the preparation and use of the scaffold. The main function of scaffolds is to offer a physical environment to regulate the healing and regeneration of injured tissues (217).

Stem cells are usually implanted into engineered scaffolds to promote tissue repair and regeneration. Mesenchymal stem cells are natural cells of tendons, so they are widely used. Mesenchymal stem cells are easy to obtain from various tissue sources. Besides, they have the characteristics of anti-inflammatory, reducing tissue inflammation, secreting a large number of trophic factors and promote tissue regeneration. That may explain why they are widely used in tendon and ligament tissue engineering (218–220). Cell secretory products such as exosomes, secretory bodies and platelet-rich plasma (PRP) can also be implanted into engineering tissues to improve the therapeutic effect (189, 221, 222).

## Conclusion

Tendon is a complex tissue with unique structure, function and mechanical properties. Damage to this vital connective tissue can lead to severe pain and disability. For this reason, improving tendon healing is an urgent need. There are many research topics in the field of tendon healing and repair. At present, gene therapy, stem cell therapy and others have made some achievements, although they are not up to the standard of clinical application. Although many other studies have focused on tendon healing, there are still many problems to be clarified about this complex process.

A recent review found a strong link between cancer and wound healing (223). Maybe we can further understand of tendon healing and thus find ways to accelerate tendon healing from some cancer studies. For example, some signs show that the temporary closure of the apoptosis regulation pathway can accelerate regular repair (223).

## Author Contributions

ZL wrote the initial manuscript and revised the manuscript. QY revised the manuscript. YZ provided initial idea of this paper and revised the manuscript. All authors contributed to the article and approved the submitted version.

## Conflict of Interest

The authors declare that the research was conducted in the absence of any commercial or financial relationships that could be construed as a potential conflict of interest.

## Publisher's Note

All claims expressed in this article are solely those of the authors and do not necessarily represent those of their affiliated organizations, or those of the publisher, the editors and the reviewers. Any product that may be evaluated in this article, or claim that may be made by its manufacturer, is not guaranteed or endorsed by the publisher.

## References

[1] Nourissat G Berenbaum F Duprez D. Tendon injury: from biology to tendon repair. Nat Rev Rheumatol. (2015) 11:223–33. 10.1038/nrrheum.2015.26 25734975

[2] Voleti PB Buckley MR Soslowsky LJ. Tendon healing: repair and regeneration. Annu Rev Biomed Eng. (2012) 14:47–71. 10.1146/annurev-bioeng-071811-150122 22809137

[3] Docheva D Müller SA Majewski M Evans CH. Biologics for tendon repair. Adv Drug Deliv Rev. (2015) 84:222–39. 10.1016/j.addr.2014.11.015 25446135 PMC4519231

[4] Bavin EP Smith O Baird AE Smith LC Guest DJ. Equine induced pluripotent stem cells have a reduced tendon differentiation capacity compared to embryonic stem cells. Front Vet Sci. (2015) 2:55. 10.3389/fvets.2015.00055 26664982 PMC4672282

[5] Molloy T Wang Y Murrell G. The roles of growth factors in tendon and ligament healing. Sports Med. (2003) 33:381–94. 10.2165/00007256-200333050-00004 12696985

[6] Hsu C Chang J. Clinical implications of growth factors in flexor tendon wound healing. J Hand Surg. (2004) 29:551–63. 10.1016/j.jhsa.2004.04.020 15249076

[7] Mienaltowski MJ Birk DE. Structure, physiology, and biochemistry of collagens. Adv Exp Med Biol. (2014) 802:5–29. 10.1007/978-94-007-7893-1_2 24443018

[8] Benjamin M Qin S Ralphs JR. Fibrocartilage associated with human tendons and their pulleys. J Anat. (1995) 187:625–33. 8586561 PMC1167465

[9] Mccormick A Charlton J Fleming D. Assessing health needs in primary care. Morbidity study from general practice provides another source of information. BMJ. (1995) 310:1534. 10.1136/bmj.310.6993.1534d 7787617 PMC2549904

[10] Riley G. Tendinopathy–from basic science to treatment. Nat Clin Pract Rheumatol. (2008) 4:82–9. 10.1038/ncprheum0700 18235537

[11] Gaida JE Alfredson H Kiss ZS Bass SL Cook JL. Asymptomatic Achilles tendon pathology is associated with a central fat distribution in men and a peripheral fat distribution in women: a crosssectional study of 298 individuals. BMC Musculoskelet Disord. (2010) 11:41. 10.1186/1471-2474-11-41 20196870 PMC2841085

[12] Maffulli N Khan KM Puddu G. Overuse tendon conditions: time to change a confusing terminology. Arthroscopy. (1998) 14:840–3. 10.1016/S0749-8063(98)70021-0 9848596

[13] Magnusson SP Langberg H Kjaer M. The pathogenesis of tendinopathy: balancing the response to loading. Nat Rev Rheumatol. (2010) 6:262–8. 10.1038/nrrheum.2010.43 20308995

[14] Magnan B Bondi M Pierantoni S Samaila E. The pathogenesis of Achilles tendinopathy: a systematic review. Foot Ankle Surg. (2014) 20:154–9. 10.1016/j.fas.2014.02.010 25103700

[15] Sharma P Maffulli N. Tendon injury and tendinopathy: healing and repair. J Bone Joint Surg Am. (2005) 87:187–202. 10.2106/00004623-200501000-00030 15634833

[16] Leadbetter WB. Cell-matrix response in tendon injury. Clin Sports Med. (1992) 11:533–78. 10.1016/S0278-5919(20)30507-X 1638640

[17] Xu Y Murrell GA. The basic science of tendinopathy. Clin Orthop Relat Res. (2008) 286:4. 10.1007/s11999-008-0286-4 PMC2505234 18478310

[18] Jelinsky SA Rodeo SA Li J Gulotta LV Archambault JM Seeherman HJ. Regulation of gene expression in human tendinopathy. BMC Musculoskelet Disord. (2011) 12:86. 10.1186/1471-2474-12-86 21539748 PMC3095578

[19] Kaux JF Forthomme B Goff CL Crielaard JM Croisier JL. Current opinions on tendinopathy. J Sports Sci Med. (2011) 10:238–53. 24149868 PMC3761855

[20] Thornton GM Hart DA. The interface of mechanical loading and biological variables as they pertain to the development of tendinosis. J Musculoskelet Neuronal Interact. (2011) 11:94–105. 21625046

[21] Wang JH Guo Q Li B. Tendon biomechanics and mechanobiology–a minireview of basic concepts and recent advancements. J Hand Ther. (2012) 25:133–40. 10.1016/j.jht.2011.07.004 21925835 PMC3244520

[22] Freedman BR Gordon JA Soslowsky LJ. The Achilles tendon: fundamental properties and mechanisms governing healing. Muscles Ligaments Tendons J. (2014) 4:245–55. 10.11138/mltj/2014.4.2.245 25332943 PMC4187594

[23] McElvany MD McGoldrick E Gee AO Neradilek MB Matsen FA 3rd. Rotator cuff repair: published evidence on factors associated with repair integrity and clinical outcome. Am J Sports Med. (2015) 43:491–500. 10.1177/0363546514529644 24753240

[24] Hope M Saxby TS. Tendon healing. Foot Ankle Clin Dec. (2007) 12:553–67. 10.1016/j.fcl.2007.07.003 17996614

[25] Guevara-Alvarez A Schmitt A Russell RP Imhoff AB Buchmann S. Growth factor delivery vehicles for tendon injuries: mesenchymal stem cells and Platelet Rich Plasma. Muscles Ligaments Tendons J. (2014) 4:378–85. 10.11138/mltj/2014.4.3.378 25489557 PMC4241431

[26] Manning CN Havlioglu N Knutsen E Sakiyama-Elbert SE Silva MJ Thomopoulos S. The early inflammatory response after flexor tendon healing: a gene expression and histological analysis. J Orthop Res. (2014) 32:645–52. 10.1002/jor.22575 24464937 PMC4076155

[27] Berglund M Hart DA Wiig M. The inflammatory response and hyaluronan synthases in the rabbit flexor tendon and tendon sheath following injury. J Hand Surg Eur. (2007) 32:581–7. 10.1016/J.JHSE.2007.05.017 17950228

[28] Massimino ML Rapizzi E Cantini M Libera LD Mazzoleni F Arslan P. ED2+ macrophages increase selectively myoblast proliferation in muscle cultures. Biochem Biophys Res Commun. (1997) 235:754–9. 10.1006/bbrc.1997.6823 9207234

[29] Gelberman RH Boyer MI Brodt MD Winters SC Silva MJ. The effect of gap formation at the repair site on the strength and excursion of intrasynovial flexor tendons. An experimental study on the early stages of tendon-healing in dogs. J Bone Joint Surg Am. (1999) 81:975–82. 10.2106/00004623-199907000-00010 10428129

[30] Manske PR. Flexor tendon healing. J Hand Surg Br. (1988) 13:237–45. 10.1016/0266-7681(88)90077-0 3049854

[31] Beredjiklian PK. Biologic aspects of flexor tendon laceration and repair. J Bone Joint Surg Am. (2003) 85:539–50. 10.2106/00004623-200303000-00025 12637445

[32] Hogan MV Bagayoko N James R Starnes T Katz A Chhabra AB. Tissue engineering solutions for tendon repair. J Am Acad Orthop Surg. (2011) 19:134–42. 10.5435/00124635-201103000-00002 21368094

[33] Sarrafian TL Wang H Hackett ES Yao JQ Shih MS Ramsay HL. Comparison of Achilles tendon repair techniques in a sheep model using a cross-linked acellular porcine dermal patch and platelet-rich plasma fibrin matrix for augmentation. J Foot Ankle Surg. (2010) 49:128–34. 10.1053/j.jfas.2009.12.005 20137980

[34] Rees JD Wilson AM Wolman RL. Current concepts in the management of tendon disorders. Rheumatology. (2006) 45:508–21. 10.1093/rheumatology/kel046 16490749

[35] Childress MA Beutler A. Management of chronic tendon injuries. Am Fam Physician. (2013) 87:486–90. 23547590

[36] Wu YF Cao Y Zhou YL Tang JB. Biomechanical comparisons of four-strand tendon repairs with double-stranded sutures: effects of different locks and suture geometry. J Hand Surg. (2011) 36:34–9. 10.1177/1753193410379554 20682582

[37] Ahmad Z Wardale J Brooks R Henson F Noorani A Rushton N. Exploring the application of stem cells in tendon repair and regeneration. Arthroscopy. (2012) 28:1018–29. 10.1016/j.arthro.2011.12.009 22381688

[38] Lee SY Kim W Lim C Chung SG. Treatment of lateral epicondylosis by using allogeneic adipose-derived mesenchymal stem cells: a pilot study. Stem Cells. (2015) 33:2995–3005. 10.1002/stem.2110 26202898

[39] Stoll LE Huang JI. Surgical treatment of distal biceps ruptures. Orthop Clin North Am. (2016) 47:189–205. 10.1016/j.ocl.2015.08.025 26614933

[40] Gilmore JH Clayton-Smith ZJ Aguilar M Pneumaticos SG Giannoudis PV. Reconstruction techniques and clinical results of patellar tendon ruptures: evidence today. Knee. (2015) 22:148–55. 10.1016/j.knee.2014.10.007 25819155

[41] Leek BT Tasto JP Tibor LM Healey RM Freemont A Linn MS. Augmentation of tendon healing with butyric acid-impregnated sutures: biomechanical evaluation in a rabbit model. Am J Sports Med. (2012) 40:1762–71. 10.1177/0363546512450691 22729622

[42] Stevenson DP Milligan SR Collins WP. Effects of platelet-derived endothelial cell growth factor/thymidine phosphorylase, substrate, and products in a three-dimensional model of angiogenesis. Am J Pathol. (1998) 152:1641–6. 9626068 PMC1858429

[43] Güleç A Türk Y Aydin BK Erkoçak ÖF Safali S Ugurluoglu C. Effect of curcumin on tendon healing: an experimental study in a rat model of Achilles tendon injury. Int Orthop. (2018) 42:1905–10. 10.1007/s00264-018-4017-5 29922838

[44] Dean BJ Lostis E Oakley T Rombach I Morrey ME Carr AJ. The risks and benefits of glucocorticoid treatment for tendinopathy: a systematic review of the effects of local glucocorticoid on tendon. Semin Arthritis Rheum. (2014) 43:570–6. 10.1016/j.semarthrit.2013.08.006 24074644

[45] Zhang K Hast MW Izumi S Usami Y Shetye S Akabudike N. Modulating glucose metabolism and lactate synthesis in injured mouse tendons: treatment with dichloroacetate, a lactate synthesis inhibitor, improves tendon healing. Am J Sports Med. (2018) 46:2222–31. 10.1177/0363546518778789 29927623 PMC6510478

[46] Tack C Shorthouse F Kass L. The physiological mechanisms of effect of vitamins and amino acids on tendon and muscle healing: a systematic review. Int J Sport Nutr Exerc Metab. (2018) 28:294–311. 10.1123/ijsnem.2017-0267 29140140

[47] Younesi M Knapik DM Cumsky J Donmez BO He P Islam A. Effects of PDGF-BB delivery from heparinized collagen sutures on the healing of lacerated chicken flexor tendon *in vivo*. Acta Biomater. (2017) 63:200–9. 10.1016/j.actbio.2017.09.006 28890257 PMC5653421

[48] James R Kesturu G Balian G Chhabra AB. Tendon: biology, biomechanics, repair, growth factors, and evolving treatment options. J Hand Surg Am. (2008) 33:102–12. 10.1016/j.jhsa.2007.09.007 18261674

[49] Titan AL Foster DS Chang J Longaker MT. Flexor tendon: development, healing, adhesion formation, and contributing growth factors. Plast Reconstr Surg. (2019) 144:639e−47e. 10.1097/PRS.0000000000006048 31568303 PMC7092377

[50] Chang J Most D Stelnicki E Siebert JW Longaker MT Hui K. Gene expression of transforming growth factor beta-1 in rabbit zone II flexor tendon wound healing: evidence for dual mechanisms of repair. Plast Reconstr Surg. (1997) 100:937–44. 10.1097/00006534-199709001-00016 9290662

[51] Chang J Thunder R Most D Longaker MT Lineaweaver WC. Studies in flexor tendon wound healing: neutralizing antibody to TGF-beta1 increases postoperative range of motion. Plast Reconstr Surg. (2000) 105:148–55. 10.1097/00006534-200001000-00025 10626983

[52] Ackerman JE Bah I Jonason JH Buckley MR Loiselle AE. Aging does not alter tendon mechanical properties during homeostasis, but does impair flexor tendon healing. J Orthop Res. (2017) 35:2716–24. 10.1002/jor.23580 28419543 PMC5645212

[53] Zhou Y Zhang L Zhao W Wu Y Zhu C Yang Y. Nanoparticle-mediated delivery of TGF-β1 miRNA plasmid for preventing flexor tendon adhesion formation. Biomaterials. (2013) 34:8269–78. 10.1016/j.biomaterials.2013.07.072 23924908

[54] Brown JP Finley VG Kuo CK. Embryonic mechanical and soluble cues regulate tendon progenitor cell gene expression as a function of developmental stage and anatomical origin. J Biomech. (2014) 47:214–22. 10.1016/j.jbiomech.2013.09.018 24231248 PMC4157071

[55] Pryce BA Watson SS Murchison ND Staverosky JA Dünker N Schweitzer R. Recruitment and maintenance of tendon progenitors by TGFbeta signaling are essential for tendon formation. Development. (2009) 136:1351–61. 10.1242/dev.027342 19304887 PMC2687466

[56] Platonova N Miquel G Chiu LY Taouji S Moroni E Colombo G. Dimerization capacities of FGF2 purified with or without heparin-affinity chromatography. PLoS ONE. (2014) 9:e110055. 10.1371/journal.pone.0110055 25299071 PMC4192534

[57] Chan BP Chan KM Maffulli N Webb S Lee KK. Effect of basic fibroblast growth factor. An *in vitro* study of tendon healing. Clin Orthop Relat Res. (1997) 342:239–47. 10.1097/00003086-199709000-00031 9308546

[58] Tsubone T Moran SL Amadio PC Zhao C An KN. Expression of growth factors in canine flexor tendon after laceration *in vivo*. Ann Plast Surg. (2004) 53:393–7. 10.1097/01.sap.0000125501.72773.01 15385778

[59] Thomopoulos S Harwood FL Silva MJ Amiel D Gelberman RH. Effect of several growth factors on canine flexor tendon fibroblast proliferation and collagen synthesis *in vitro*. J Hand Surg Am. (2005) 30:441–7. 10.1016/j.jhsa.2004.12.006 15925149

[60] Thomopoulos S Das R Silva MJ Sakiyama-Elbert S Harwood FL Zampiakis E. Enhanced flexor tendon healing through controlled delivery of PDGF-BB. J Orthop Res. (2009) 27:1209–15. 10.1002/jor.20875 19322789 PMC2916020

[61] Thomopoulos S Kim HM Das R Silva MJ Sakiyama-Elbert S Amiel D. The effects of exogenous basic fibroblast growth factor on intrasynovial flexor tendon healing in a canine model. J Bone Joint Surg Am. (2010) 92:2285–93. 10.2106/JBJS.I.01601 20926722 PMC2945931

[62] Tang JB Wu YF Cao Y Chen CH Zhou YL Avanessian B. Basic FGF or VEGF gene therapy corrects insufficiency in the intrinsic healing capacity of tendons. Sci Rep. (2016) 6:20643. 10.1038/srep20643 26865366 PMC4749961

[63] Thomopoulos S Das R Sakiyama-Elbert S Silva MJ Charlton N Gelberman RH. bFGF and PDGF-BB for tendon repair: controlled release and biologic activity by tendon fibroblasts *in vitro*. Ann Biomed Eng. (2010) 38:225–34. 10.1007/s10439-009-9844-5 19937274 PMC2843401

[64] Shibuya M VEGFR. and type-V RTK activation and signaling. Cold Spring Harb Perspect Biol. (2013) 5:a009092. 10.1101/cshperspect.a009092 24086040 PMC3783052

[65] Petersen W Pufe T Kurz B Mentlein R Tillmann B. Angiogenesis in fetal tendon development: spatial and temporal expression of the angiogenic peptide vascular endothelial cell growth factor. Anat Embryol. (2002) 205:263–70. 10.1007/s00429-002-0241-1 12136256

[66] Pufe T Petersen WJ Mentlein R Tillmann BN. The role of vasculature and angiogenesis for the pathogenesis of degenerative tendons disease. Scand J Med Sci Sports. (2005) 15:211–22. 10.1111/j.1600-0838.2005.00465.x 15998338

[67] Scott A Danielson P. An emerging role for angiogenesis in tendinopathy. Eur Musculoskelet Rev. (2009) 4:75–6. 26213568 PMC4509735

[68] Gelberman RH Steinberg D Amiel D Akeson W. Fibroblast chemotaxis after tendon repair. J Hand Surg Am. (1991) 16:686–93. 10.1016/0363-5023(91)90195-H 1880367

[69] Bidder M Towler DA Gelberman RH Boyer MI. Expression of mRNA for vascular endothelial growth factor at the repair site of healing canine flexor tendon. J Orthop Res. (2000) 18:247–52. 10.1002/jor.1100180212 10815825

[70] Boyer MI Watson JT Lou J Manske PR Gelberman RH Cai SR. Quantitative variation in vascular endothelial growth factor mRNA expression during early flexor tendon healing: an investigation in a canine model. J Orthop Res. (2001) 19:869–72. 10.1016/S0736-0266(01)00017-1 11562135

[71] Raica M Cimpean AM. Platelet-derived growth factor (PDGF)/PDGF receptors (PDGFR) axis as target for antitumor and antiangiogenic therapy. Pharmaceuticals. (2010) 3:572–99. 10.3390/ph3030572 27713269 PMC4033970

[72] Sugg KB Markworth JF Disser NP Rizzi AM Talarek JR Sarver DC. Postnatal tendon growth and remodeling require platelet-derived growth factor receptor signaling. Am J Physiol Cell Physiol. (2018) 314:C389–403. 10.1152/ajpcell.00258.2017 29341790 PMC5966786

[73] Scherping SC Jr Schmidt CC Georgescu HI Kwoh CK Evans CH Woo SL. Effect of growth factors on the proliferation of ligament fibroblasts from skeletally mature rabbits. Connect Tissue Res. (1997) 36:1–8. 10.3109/03008209709160209 9298619

[74] Thomopoulos S Zaegel M Das R Harwood FL Silva MJ Amiel D. released in tendon repair using a novel delivery system promotes cell proliferation and collagen remodeling. J Orthop Res. (2007) 25:1358–68. 10.1002/jor.20444 17551975

[75] Hee CK Dines JS Dines DM Roden CM Wisner-Lynch LA Turner AS. Augmentation of a rotator cuff suture repair using rhPDGF-BB and a type I bovine collagen matrix in an ovine model. Am J Sports Med. (2011) 39:1630–9. 10.1177/0363546511404942 21555508

[76] Hee CK Dines JS Solchaga LA Shah VR Hollinger JO. Regenerative tendon and ligament healing: opportunities with recombinant human platelet-derived growth factor BB-homodimer. Tissue Eng Part B Rev. (2012) 18:225–34. 10.1089/ten.teb.2011.0603 22145770

[77] Suwalski A Dabboue H Delalande A Bensamoun SF Canon F Midoux P. Accelerated Achilles tendon healing by PDGF gene delivery with mesoporous silica nanoparticles. Biomaterials. (2010) 31:5237–45. 10.1016/j.biomaterials.2010.02.077 20334910

[78] Madrigal JL Stilhano R Silva EA. Biomaterial-guided gene delivery for musculoskeletal tissue repair. Tissue Eng B Rev. (2017) 23:347–61. 10.1089/ten.teb.2016.0462 28166711 PMC5749599

[79] Liu S Hu C Li F Li XJ Cui W Fan C. Prevention of peritendinous adhesions with electrospun ibuprofen-loaded poly(L-lactic acid)-polyethylene glycol fibrous membranes. Tissue Eng A. (2013) 19:529–37. 10.1089/ten.tea.2012.0208 23013368

[80] Branford OA Klass BR Grobbelaar AO Rolfe KJ. The growth factors involved in flexor tendon repair and adhesion formation. J Hand Surg. (2014) 39:60–70. 10.1177/1753193413509231 24162452

[81] Tang JB Chang J Elliot D Lalonde DH Sandow M Vögelin E. Flexor Tendon Committee report 2014: from the IFSSH Flexor Tendon Committee (Chairman: Jin Bo Tang). J Hand Surg. (2014) 39:107–15. 10.1177/1753193413500768 23962872

[82] Karaaltin MV Ozalp B Dadaci M Kayikcioglu A Kecik A Oner F. The effects of 5-fluorouracil on flexor tendon healing by using a biodegradable gelatin, slow releasing system: experimental study in a hen model. J Hand Surg Eur. (2013) 38:651–7. 10.1177/1753193412458646 22918883

[83] Wu YF Tang JB. Recent developments in flexor tendon repair techniques and factors influencing strength of the tendon repair. J Hand Surg Eur. (2014) 39:6–19. 10.1177/1753193413492914 23792441

[84] Chen G Zhang S Zhang Z. Over-expression of has2 in synovium-derived mesenchymal stem cells may prevent adhesions following surgery of the digital flexor tendons. Med Hypotheses. (2011) 76:314–6. 10.1016/j.mehy.2010.09.022 21084166

[85] Patil S Gao YG Lin X Li Y Dang K Tian Y. The development of functional non-viral vectors for gene delivery. Int J Mol Sci. (2019) 20:5491. 10.3390/ijms20215491 31690044 PMC6862238

[86] Tang JB Zhou YL Wu YF Liu PY Wang XT. Gene therapy strategies to improve strength and quality of flexor tendon healing. Expert Opin Biol Ther. (2016) 16:291–301. 10.1517/14712598.2016.1134479 26853840

[87] Madry H Orth P Cucchiarini M. Gene therapy for cartilage repair. Cartilage. (2011) 2:201–25. 10.1177/1947603510392914 26069580 PMC4300805

[88] Farini A Sitzia C Erratico S Meregalli M Torrente Y. Influence of immune responses in gene/stem cell therapies for muscular dystrophies. Biomed Res Int. (2014) 2014:818107. 10.1155/2014/818107 24959590 PMC4052166

[89] Patil SD Rhodes DG Burgess DJ. DNA-based therapeutics and DNA delivery systems: a comprehensive review. AAPS J. (2005) 7:E61–77. 10.1208/aapsj070109 16146351 PMC2751499

[90] Thierry AR Vives E Richard JP Prevot P Martinand-Mari C Robbins I. Cellular uptake and intracellular fate of antisense oligonucleotides. Curr Opin Mol Ther. (2003) 5:133–8. 10.1016/0962-8924(92)90100-2 12772502

[91] Dai Q Manfield L Wang Y Murrell GA. Adenovirus-mediated gene transfer to healing tendon–enhanced efficiency using a gelatin sponge. J Orthop Res. (2003) 21:604–9. 10.1016/S0736-0266(02)00239-5 12798058

[92] Wong SP Argyros O Harbottle RP. Vector systems for prenatal gene therapy: principles of non-viral vector design and production. Methods Mol Biol. (2012) 891:133–67. 10.1007/978-1-61779-873-3_7 22648771

[93] Xiao W Chen X Yang L Mao Y Wei Y Chen L. Co-delivery of doxorubicin and plasmid by a novel FGFR-mediated cationic liposome. Int J Pharm. (2010) 393:119–26. 10.1016/j.ijpharm.2010.04.018 20416367

[94] Tang JB Cao Y Zhu B Xin KQ Wang XT Liu PY. Adeno-associated virus-2-mediated bFGF gene transfer to digital flexor tendons significantly increases healing strength. An *in vivo* study. J Bone Joint Surg Am. (2008) 90:1078–89. 10.2106/JBJS.F.01188 18451401

[95] Chira S Jackson CS Oprea I Ozturk F Pepper MS Diaconu I. Progresses towards safe and efficient gene therapy vectors. Oncotarget. (2015) 6:30675–703. 10.18632/oncotarget.5169 26362400 PMC4741561

[96] Xu H Li Z Si J. Nanocarriers in gene therapy: a review. J Biomed Nanotechnol. (2014) 10:3483–507. 10.1166/jbn.2014.2044 26000367

[97] Itaka K Kataoka K. Recent development of non-viral gene delivery systems with virus-like structures and mechanisms. Eur J Pharm Biopharm. (2009) 71:475–83. 10.1016/j.ejpb.2008.09.019 18955136

[98] Brenner M. Gene transfer by adenovectors. Blood. (1999) 94:3965–7. 10.1182/blood.V94.12.3965.424k42_3965_3967 10590039

[99] Wang W Li W Ma N Steinhoff G. Non-viral gene delivery methods. Curr Pharm Biotechnol. (2013) 14:46–60. 10.2174/1389201011314010008 23437936

[100] Bolt P Clerk AN Luu HH Kang Q Kummer JL Deng ZL. BMP-14 gene therapy increases tendon tensile strength in a rat model of Achilles tendon injury. J Bone Joint Surg Am. (2007) 89:1315–20. 10.2106/00004623-200706000-00021 17545436

[101] Wang XT Liu PY Xin KQ Tang JB. Tendon healing *in vitro*: bFGF gene transfer to tenocytes by adeno-associated viral vectors promotes expression of collagen genes. J Hand Surg Am. (2005) 30:1255–61. 10.1016/j.jhsa.2005.06.001 16344185

[102] Uggen JC Dines J Uggen CW Mason JS Razzano P Dines D. Tendon gene therapy modulates the local repair environment in the shoulder. J Am Osteopath Assoc. (2005) 105:20–1. 15710662

[103] Waehler R Russell SJ Curiel DT. Engineering targeted viral vectors for gene therapy. Nat Rev Genet. (2007) 8:573–87. 10.1038/nrg2141 17607305 PMC7097627

[104] Katz MG Fargnoli AS Kendle AP Hajjar RJ Bridges CR. Gene therapy in cardiac surgery: clinical trials, challenges, and perspectives. Ann Thorac Surg. (2016) 101:2407–16. 10.1016/j.athoracsur.2015.12.004 26801060 PMC4987708

[105] Li Y Li B Li CJ Li LJ. Key points of basic theories and clinical practice in rAd-p53 (Gendicine™) gene therapy for solid malignant tumors. Expert Opin Biol Ther. (2015) 15:437–54. 10.1517/14712598.2015.990882 25496374

[106] Sheridan C. Gene therapy finds its niche [published correction appears in Nat Biotechnol. 2011 May;29:459]. Nat Biotechnol. (2011) 29:121–8. 10.1038/nbt.1769 21301435

[107] Leen AM Christin A Khalil M Weiss H Gee AP Brenner MK. Identification of hexon-specific CD4 and CD8 T-cell epitopes for vaccine and immunotherapy. J Virol. (2008) 82:546–54. 10.1128/JVI.01689-07 17942545 PMC2224388

[108] Choi JW Kang E Kwon OJ Yun TJ Park HK Kim PH. Local sustained delivery of oncolytic adenovirus with injectable alginate gel for cancer virotherapy. Gene Ther. (2013) 20:880–92. 10.1038/gt.2013.10 23514707

[109] Bertoni C Jarrahian S Wheeler TM Li Y Olivares EC Calos MP Rando TA. Enhancement of plasmid-mediated gene therapy for muscular dystrophy by directed plasmid integration. Proc Natl Acad Sci USA. (2006) 103:419–24. 10.1073/pnas.0504505102 16387861 PMC1326153

[110] Zhou Y Zhu C Wu YF Zhang L Tang JB. Effective modulation of transforming growth factor-β1 expression through engineered microRNA-based plasmid-loaded nanospheres. Cytotherapy. (2015) 17:320–9. 10.1016/j.jcyt.2014.09.004 25457276

[111] Smith PJ Giroud M Wiggins HL Gower F Thorley JA Stolpe B. Cellular entry of nanoparticles via serum sensitive clathrin-mediated endocytosis, and plasma membrane permeabilization. Int J Nanomedicine. (2012) 7:2045–55. 10.2147/IJN.S29334 22619541 PMC3356167

[112] Panyam J Zhou WZ Prabha S Sahoo SK Labhasetwar V. Rapid endo-lysosomal escape of poly(DL-lactide-co-glycolide) nanoparticles: implications for drug and gene delivery. FASEB J. (2002) 16:1217–26. 10.1096/fj.02-0088com 12153989

[113] Merkel OM Beyerle A Beckmann BM Zheng M Hartmann RK Stöger T. Polymer-related off-target effects in non-viral siRNA delivery. Biomaterials. (2011) 32:2388–98. 10.1016/j.biomaterials.2010.11.081 21183213

[114] Gao YG Alam U Tang Q Shi YD Zhang Y Wang R. Functional lipids based on [12]aneN3 and naphthalimide as efficient non-viral gene vectors. Org Biomol Chem. (2016) 14:6346–54. 10.1039/C6OB00917D 27273411

[115] Qadir A Gao Y Suryaji P Tian Y Lin X Dang K. Non-viral delivery system and targeted bone disease therapy. Int J Mol Sci. (2019) 20:565. 10.3390/ijms20030565 30699924 PMC6386958

[116] Chattopadhyay S Raines RT. Review collagen-based biomaterials for wound healing. Biopolymers. (2014) 101:821–33. 10.1002/bip.22486 24633807 PMC4203321

[117] Smeets NM Bakaic E Patenaude M Hoare T. Injectable and tunable poly(ethylene glycol) analogue hydrogels based on poly(oligoethylene glycol methacrylate). Chem Commun. (2014) 50:3306–9. 10.1039/c3cc48514e 24531402

[118] Gentile P Chiono V Carmagnola I Hatton PV. An overview of poly(lactic-co-glycolic) acid (PLGA)-based biomaterials for bone tissue engineering. Int J Mol Sci. (2014) 15:3640–59. 10.3390/ijms15033640 24590126 PMC3975359

[119] Dash TK Konkimalla VB. Poly-?-caprolactone based formulations for drug delivery and tissue engineering: a review. J Control Release. (2012) 158:15–33. 10.1016/j.jconrel.2011.09.064 21963774

[120] Zu Y Huang S Liao WC Lu Y Wang S. Gold nanoparticles enhanced electroporation for mammalian cell transfection. J Biomed Nanotechnol. (2014) 10:982–92. 10.1166/jbn.2014.1797 24749393 PMC4696415

[121] Torio-Padron N Borges J Momeni A Mueller MC Tegtmeier FT Stark GB. Implantation of VEGF transfected preadipocytes improves vascularization of fibrin implants on the cylinder chorioallantoic membrane (CAM) model. Minim Invasive Ther Allied Technol. (2007) 16:155–62. 10.1080/13645700701384116 17573620

[122] Kimelman-Bleich N Pelled G Zilberman Y Kallai I Mizrahi O Tawackoli W. Targeted gene-and-host progenitor cell therapy for non-union bone fracture repair. Mol Ther. (2011) 19:53–9. 10.1038/mt.2010.190 20859259 PMC3017436

[123] Li YH Shi QS Du J Jin LF Du LF Liu PF. Targeted delivery of biodegradable nanoparticles with ultrasound-targeted microbubble destruction-mediated hVEGF-siRNA transfection in human PC-3 cells *in vitro*. Int J Mol Med. (2013) 31:163–71. 10.3892/ijmm.2012.1175 23138749

[124] Nomikou N Feichtinger GA Redl H McHale AP. Ultrasound-mediated gene transfer (sonoporation) in fibrin-based matrices: potential for use in tissue regeneration. J Tissue Eng Regen Med. (2016) 10:29–39. 10.1002/term.1730 23596105

[125] Sun L Li H Qu L Zhu R Fan X Xue Y. Immobilized lentivirus vector on chondroitin sulfate-hyaluronate acid-silk fibroin hybrid scaffold for tissue-engineered ligament-bone junction. Biomed Res Int. (2014) 2014:816979. 10.1155/2014/816979 25019087 PMC4075190

[126] Rey-Rico A Cucchiarini M. Controlled release strategies for rAAV-mediated gene delivery. Acta Biomater. (2016) 29:1–10. 10.1016/j.actbio.2015.10.015 26472612

[127] Hou Y Mao Z Wei X Lin L Chen L Wang H. Effects of transforming growth factor-beta1 and vascular endothelial growth factor 165 gene transfer on Achilles tendon healing. Matrix Biol. (2009) 28:324–35. 10.1016/j.matbio.2009.04.007 19389474

[128] Chen X Yin Z Chen JL Liu HH Shen WL Fang Z. Scleraxis-overexpressed human embryonic stem cell-derived mesenchymal stem cells for tendon tissue engineering with knitted silk-collagen scaffold. Tissue Eng A. (2014) 20:1583–92. 10.1089/ten.tea.2012.0656 24328506

[129] Tian F Ji XL Xiao WA Wang B Wang F. CXCL13 promotes the effect of bone marrow mesenchymal stem cells (MSCs) on tendon-bone healing in rats and in C3HIOT1/2 cells. Int J Mol Sci. (2015) 16:3178–87. 10.3390/ijms16023178 25647417 PMC4346887

[130] Delalande A Gosselin MP Suwalski A Guilmain W Leduc C Berchel M. Enhanced Achilles tendon healing by fibromodulin gene transfer. Nanomedicine. (2015) 11:1735–44. 10.1016/j.nano.2015.05.004 26048315

[131] Gao Y Zhang Y Lu Y Wang Y Kou X Lou Y. TOB1 Deficiency enhances the effect of bone marrow-derived mesenchymal stem cells on tendon-bone healing in a rat rotator cuff repair model. Cell Physiol Biochem. (2016) 38:319–29. 10.1159/000438632 26824451

[132] Jiang Y Shi Y He J Zhang Z Zhou G Zhang W. Enhanced tenogenic differentiation and tendon-like tissue formation by tenomodulin overexpression in murine mesenchymal stem cells. J Tissue Eng Regen Med. (2017) 11:2525–36. 10.1002/term.2150 27098985

[133] Yang QQ Shao YX Zhang LZ Zhou YL. Therapeutic strategies for flexor tendon healing by nanoparticle-mediated co-delivery of bFGF and VEGFA genes. Colloids Surf B Biointerfaces. (2018) 164:165–76. 10.1016/j.colsurfb.2018.01.031 29413593

[134] Zhou YL Yang QQ Yan YY Zhang L Wang QH Ju F. Gene-loaded nanoparticle-coated sutures provide effective gene delivery to enhance tendon healing. Mol Ther. (2019) 27:1534–46. 10.1016/j.ymthe.2019.05.024 31278034 PMC6731186

[135] Ejtehadifar M Shamsasenjan K Movassaghpour A Akbarzadehlaleh P Dehdilani N Abbasi P. The effect of hypoxia on mesenchymal stem cell biology. Adv Pharm Bull. (2015) 5:141–9. 10.15171/apb.2015.021 26236651 PMC4517092

[136] Wu T Liu Y Wang B Sun Y Xu J Yuk-Wai LW. The use of cocultured mesenchymal stem cells with tendon-derived stem cells as a better cell source for tendon repair. Tissue Eng A. (2016) 22:1229–40. 10.1089/ten.tea.2016.0248 27609185

[137] Gonçalves AI Rotherham M Markides H Rodrigues MT Reis RL Gomes ME. Triggering the activation of Activin A type II receptor in human adipose stem cells towards tenogenic commitment using mechanomagnetic stimulation. Nanomedicine. (2018) 14:1149–59. 10.1016/j.nano.2018.02.008 29471171

[138] Chen E Yang L Ye C Zhang W Ran J Xue D. An asymmetric chitosan scaffold for tendon tissue engineering: *in vitro* and *in vivo* evaluation with rat tendon stem/progenitor cells. Acta Biomater. (2018) 73:377–87. 10.1016/j.actbio.2018.04.027 29678676

[139] Rajpar I Barrett JG. Optimizing growth factor induction of tenogenesis in three-dimensional culture of mesenchymal stem cells. J Tissue Eng. (2019) 10:2041731419848776. 10.1177/2041731419848776 31205672 PMC6535701

[140] Slone HS Romine SE Premkumar A Xerogeanes JW. Quadriceps tendon autograft for anterior cruciate ligament reconstruction: a comprehensive review of current literature and systematic review of clinical results. Arthroscopy. (2015) 31:541–54. 10.1016/j.arthro.2014.11.010 25543249

[141] Hollawell S Baione W. Chronic Achilles tendon rupture reconstructed with Achilles tendon allograft and xenograft combination. J Foot Ankle Surg. (2015) 54:1146–50. 10.1053/j.jfas.2014.09.006 25488191

[142] Güngörmüş C Kolankaya D Aydin E. Histopathological and biomechanical evaluation of tenocyte seeded allografts on rat Achilles tendon regeneration. Biomaterials. (2015) 51:108–18. 10.1016/j.biomaterials.2015.01.077 25771002

[143] Liao JCY He M Gan AWT Wen F Tan LP Chong AKS. The effects of bi-functional anti-adhesion scaffolds on flexor tendon healing in a rabbit model. J Biomed Mater Res B Appl Biomater. (2018) 106:2605–14. 10.1002/jbm.b.34077 29424966

[144] Wang W Zhao J Yao Z Liu J Shi Z Li Y. Oriented inner fabrication of bi-layer biomimetic tendon sheath for anti-adhesion and tendon healing. Ther Adv Chronic Dis. (2020) 11:2040622320944779. 10.1177/2040622320944779 32821363 PMC7412925

[145] Abat F Alfredson H Cucchiarini M Madry H Marmotti A Mouton C. Current trends in tendinopathy: consensus of the ESSKA basic science committee. Part II: treatment options. J Exp Orthop. (2018) 5:38. 10.1186/s40634-018-0145-5 30251203 PMC6153202

[146] Losi P Briganti E Errico C Lisella A Sanguinetti E Chiellini F. Fibrin-based scaffold incorporating VEGF- and bFGF-loaded nanoparticles stimulates wound healing in diabetic mice. Acta Biomater. (2013) 9:7814–21. 10.1016/j.actbio.2013.04.019 23603001

[147] Nauta A Seidel C Deveza L Montoro D Grova M Ko SH. Adipose-derived stromal cells overexpressing vascular endothelial growth factor accelerate mouse excisional wound healing. Mol Ther. (2013) 21:445–55. 10.1038/mt.2012.234 23164936 PMC3594010

[148] Migliorini F Rath B Tingart M Baroncini A Quack V Eschweiler J. Autogenic mesenchymal stem cells for intervertebral disc regeneration. Int Orthop. (2019) 43:1027–36. 10.1007/s00264-018-4218-y 30415465

[149] Migliorini F Berton A Salvatore G Candela V Khan W Longo UG. Autologous chondrocyte implantation and mesenchymal stem cells for the treatments of chondral defects of the knee- a systematic review. Curr Stem Cell Res Ther. (2020) 15:547–56. 10.2174/1574888X15666200221122834 32081109

[150] Centeno C Sheinkop M Dodson E Stemper I Williams C Hyzy M. specific protocol of autologous bone marrow concentrate and platelet products versus exercise therapy for symptomatic knee osteoarthritis: a randomized controlled trial with 2 year follow-up. J Transl Med. (2018) 16:355. 10.1186/s12967-018-1736-8 30545387 PMC6293635

[151] Law L Hunt CL van Wijnen AJ Nassr A Larson AN Eldrige JS. Office-based mesenchymal stem cell therapy for the treatment of musculoskeletal disease: a systematic review of recent human studies. Pain Med. (2019) 20:1570–83. 10.1093/pm/pny256 30597057

[152] Tetta C Consiglio AL Bruno S Tetta E Gatti E Dobreva M. Camussi G. The role of microvesicles derived from mesenchymal stem cells in tissue regeneration; a dream for tendon repair? Muscles Ligaments Tendons J. (2012) 2:212–21. 23738299 PMC3666529

[153] Andia I Maffulli N. Biological therapies in regenerative sports medicine. Sports Med. (2017) 47:807–28. 10.1007/s40279-016-0620-z 27677916

[154] Lui PP Wong OT. Tendon stem cells: experimental and clinical perspectives in tendon and tendon-bone junction repair. Muscles Ligaments Tendons J. (2012) 2:163–8. 23738293 PMC3666522

[155] Chu CR Rodeo S Bhutani N Goodrich LR Huard J Irrgang J. Optimizing clinical use of biologics in orthopaedic surgery: consensus recommendations from the 2018 AAOS/NIH U-13 Conference. J Am Acad Orthop Surg. (2019) 27:e50–63. 10.5435/JAAOS-D-18-00305 30300216 PMC6314629

[156] Durant TJ Dyment N McCarthy MB Cote MP Arciero RA Mazzocca AD. Mesenchymal stem cell response to growth factor treatment and low oxygen tension in 3-dimensional construct environment. Muscles Ligaments Tendons J. (2014) 4:46–51. 10.32098/mltj.01.2014.09 24932447 PMC4049650

[157] Filomeno P Dayan V Touriño C. Stem cell research and clinical development in tendon repair. Muscles Ligaments Tendons J. (2012) 2:204–11. 23738298 PMC3666527

[158] Nichols AEC Best KT Loiselle AE. The cellular basis of fibrotic tendon healing: challenges and opportunities. Transl Res. (2019) 209:156–68. 10.1016/j.trsl.2019.02.002 30776336 PMC6545261

[159] Galatz LM Gerstenfeld L Heber-Katz E Rodeo SA. Tendon regeneration and scar formation: the concept of scarless healing. J Orthop Res. (2015) 33:823–31. 10.1002/jor.22853 25676657 PMC6084432

[160] Shojaee A Parham A. Strategies of tenogenic differentiation of equine stem cells for tendon repair: current status and challenges. Stem Cell Res Ther. (2019) 10:181. 10.1186/s13287-019-1291-0 31215490 PMC6582602

[161] Arany PR Cho A Hunt TD Sidhu G Shin K Hahm E. Photoactivation of endogenous latent transforming growth factor-β1 directs dental stem cell differentiation for regeneration. Sci Transl Med. (2014) 6:238ra69. 10.1126/scitranslmed.3008234 24871130 PMC4113395

[162] Chen FM Wu LA Zhang M Zhang R Sun HH. Homing of endogenous stem/progenitor cells for *in situ* tissue regeneration: promises, strategies, and translational perspectives. Biomaterials. (2011) 32:3189–209. 10.1016/j.biomaterials.2010.12.032 21300401

[163] Miller FD. Kaplan DR. Mobilizing endogenous stem cells for repair and regeneration: are we there yet? Cell Stem Cell. (2012) 10:650–2. 10.1016/j.stem.2012.05.004 22704501

[164] Vanden Berg-Foels WS. *In situ* tissue regeneration: chemoattractants for endogenous stem cell recruitment. Tissue Eng Part B Rev. (2014) 20:28–39. 10.1089/ten.teb.2013.0100 23678952 PMC3922297

[165] Martins IJ. Anti-aging genes improve appetite regulation and reverse cell senescence and apoptosis in global populations. Adv Aging Res. (2016) 5:9–26. 10.4236/aar.2016.51002

[166] Martins IJ. Single gene inactivation with implications to diabetes and multiple organ dysfunction syndrome. J Clin Epigenet. (2017) 2017:100058. 10.21767/2472-1158.100058

[167] Martins IJ. Functional foods and active molecules with relevance to health and chronic disease. Funct Foods Health Dis. (2017) 7:833–6. 10.31989/ffhd.v7i10.387

[168] Liu J Han W Chen L Tang K. Mechanism of osteogenic and adipogenic differentiation of tendon stem cells induced by sirtuin 1. Mol Med Rep. (2016) 14:1643–8. 10.3892/mmr.2016.5417 27357961

[169] Fodor WL. Tissue engineering and cell based therapies, from the bench to the clinic: the potential to replace, repair and regenerate. Reprod Biol Endocrinol. (2003) 1:102. 10.1186/1477-7827-1-102 14614775 PMC293418

[170] Lee CH Cook JL Mendelson A Moioli EK Yao H Mao JJ. Regeneration of the articular surface of the rabbit synovial joint by cell homing: a proof of concept study. Lancet. (2010) 376:440–8. 10.1016/S0140-6736(10)60668-X 20692530 PMC4035014

[171] Mao JJ Giannobile WV Helms JA Hollister SJ Krebsbach PH Longaker MT. Craniofacial tissue engineering by stem cells. J Dent Res. (2006) 85:966–79. 10.1177/154405910608501101 17062735 PMC2571078

[172] Prockop DJ. Repair of tissues by adult stem/progenitor cells (MSCs): controversies, myths, and changing paradigms. Mol Ther. (2009) 17:939–46. 10.1038/mt.2009.62 19337235 PMC2835176

[173] Zakrzewski W Dobrzyński M Szymonowicz M Rybak Z. Stem cells: past, present, and future. Stem Cell Res Ther. (2019) 10:68. 10.1186/s13287-019-1165-5 30808416 PMC6390367

[174] Kean TJ Lin P Caplan AI Dennis JE. MSCs: delivery routes and engraftment, cell-targeting strategies, and immune modulation. Stem Cells Int. (2013) 2013:732742. 10.1155/2013/732742 24000286 PMC3755386

[175] Järvinen M Józsa L Kannus P Järvinen TL Kvist M Leadbetter W. Histopathological findings in chronic tendon disorders. Scand J Med Sci Sports. (1997) 7:86–95. 10.1111/j.1600-0838.1997.tb00124.x 9211609

[176] Järvinen TA Ruoslahti E. Molecular changes in the vasculature of injured tissues. Am J Pathol. (2007) 171:702–11. 10.2353/ajpath.2007.061251 17600129 PMC1934529

[177] Järvinen TA Ruoslahti E. Target-seeking antifibrotic compound enhances wound healing and suppresses scar formation in mice. Proc Natl Acad Sci USA. (2010) 107:21671–6. 10.1073/pnas.1016233107 21106754 PMC3003105

[178] Toba M Alzoubi A O'Neill K Abe K Urakami T Komatsu M. A novel vascular homing peptide strategy to selectively enhance pulmonary drug efficacy in pulmonary arterial hypertension. Am J Pathol. (2014) 184:369–75. 10.1016/j.ajpath.2013.10.008 24401613 PMC3906494

[179] Kean TJ Duesler L Young RG Dadabayev A Olenyik A Penn M. Development of a peptide-targeted, myocardial ischemia-homing, mesenchymal stem cell. J Drug Target. (2012) 20:23–32. 10.3109/1061186X.2011.622398 22047107 PMC9208672

[180] Järvinen TAH Rashid J Valmari T May U Ahsan F. Systemically administered, target-specific therapeutic recombinant proteins and nanoparticles for regenerative medicine. ACS Biomater Sci Eng. (2017) 3:1273–82. 10.1021/acsbiomaterials.6b00746 33440515

[181] Caplan AI Correa D. The MSC: an injury drugstore. Cell Stem Cell. (2011) 9:11–5. 10.1016/j.stem.2011.06.008 21726829 PMC3144500

[182] Omi R Gingery A Steinmann SP Amadio PC An KN Zhao C. Rotator cuff repair augmentation in a rat model that combines a multilayer xenograft tendon scaffold with bone marrow stromal cells. J Shoulder Elbow Surg. (2016) 25:469–77. 10.1016/j.jse.2015.08.008 26387915 PMC5175472

[183] Marmotti A de Girolamo L Bonasia DE Bruzzone M Mattia S Rossi R. Bone marrow derived stem cells in joint and bone diseases: a concise review. Int Orthop. (2014) 38:1787–801. 10.1007/s00264-014-2445-4 25005462

[184] Pascucci L Alessandri G. Dall'Aglio C Mercati F Coliolo P Bazzucchi C. Membrane vesicles mediate pro-angiogenic activity of equine adipose-derived mesenchymal stromal cells. Vet J. (2014) 202:361–6. 10.1016/j.tvjl.2014.08.021 25241947

[185] Burk J Gittel C Heller S Pfeiffer B Paebst F Ahrberg AB. Gene expression of tendon markers in mesenchymal stromal cells derived from different sources. BMC Res Notes. (2014) 7:826. 10.1186/1756-0500-7-826 25412928 PMC4247609

[186] Youngstrom DW LaDow JE Barrett JG. Tenogenesis of bone marrow-, adipose-, and tendon-derived stem cells in a dynamic bioreactor. Connect Tissue Res. (2016) 57:454–65. 10.3109/03008207.2015.1117458 27028488

[187] Grier WK Iyoha EM Harley BAC. The influence of pore size and stiffness on tenocyte bioactivity and transcriptomic stability in collagen-GAG scaffolds. J Mech Behav Biomed Mater. (2017) 65:295–305. 10.1016/j.jmbbm.2016.08.034 27614271 PMC5154820

[188] Yang J Zhao Q Wang K Liu H Ma C Huang H. Isolation and biological characterization of tendon-derived stem cells from fetal bovine. In vitro Cell Dev Biol Anim. (2016) 52:846–56. 10.1007/s11626-016-0043-z 27130678 PMC5023758

[189] Popov C Kohler J Docheva D. Activation of EphA4 and EphB2 reverse signaling restores the age-associated reduction of self-renewal, migration, and actin turnover in human tendon stem/progenitor cells. Front Aging Neurosci. (2016) 7:246. 10.3389/fnagi.2015.00246 26779014 PMC4701947

[190] Ruzzini L Abbruzzese F Rainer A Longo UG Trombetta M Maffulli N. Characterization of age-related changes of tendon stem cells from adult human tendons. Knee Surg Sports Traumatol Arthrosc. (2014) 22:2856–66. 10.1007/s00167-013-2457-4 23503946

[191] Giai Via A McCarthy MB de Girolamo L Ragni E Oliva F Maffulli N. Making them commit: strategies to influence phenotypic differentiation in mesenchymal stem cells. Sports Med Arthrosc Rev. (2018) 26:64–9. 10.1097/JSA.0000000000000187 29722765

[192] Ekwueme EC Shah JV Mohiuddin M Ghebes CA Crispim JF Saris DB. Cross-talk between human tenocytes and bone marrow stromal cells potentiates extracellular matrix remodeling *in vitro*. J Cell Biochem. (2016) 117:684–93. 10.1002/jcb.25353 26308651 PMC4943840

[193] Bi Y Ehirchiou D Kilts TM Inkson CA Embree MC Sonoyama W. Identification of tendon stem/progenitor cells and the role of the extracellular matrix in their niche. Nat Med. (2007) 13:1219–27. 10.1038/nm1630 17828274

[194] Vermeulen S Vasilevich A Tsiapalis D Roumans N Vroemen P Beijer NRM. Identification of topographical architectures supporting the phenotype of rat tenocytes. Acta Biomater. (2019) 83:277–90. 10.1016/j.actbio.2018.10.041 30394345

[195] Guest DJ Smith MR Allen WR. Equine embryonic stem-like cells and mesenchymal stromal cells have different survival rates and migration patterns following their injection into damaged superficial digital flexor tendon. Equine Vet J. (2010) 42:636–42. 10.1111/j.2042-3306.2010.00112.x 20840579

[196] Saito S Ugai H Sawai K Yamamoto Y Minamihashi A Kurosaka K. Isolation of embryonic stem-like cells from equine blastocysts and their differentiation *in vitro*. FEBS Lett. (2002) 531:389–96. 10.1016/S0014-5793(02)03550-0 12435581

[197] Watts AE Yeager AE Kopyov OV Nixon AJ. Fetal derived embryonic-like stem cells improve healing in a large animal flexor tendonitis model. Stem Cell Res Ther. (2011) 2:4. 10.1186/scrt45 21272343 PMC3092144

[198] Citeroni MR Ciardulli MC Russo V Della Porta G Mauro A El Khatib M. *In vitro* innovation of tendon tissue engineering strategies. Int J Mol Sci. (2020) 21:6726. 10.3390/ijms21186726 32937830 PMC7555358

[199] Pas HIMFL Moen MH Haisma HJ Winters M. No evidence for the use of stem cell therapy for tendon disorders: a systematic review. Br J Sports Med. (2017) 51:996–1002. 10.1136/bjsports-2016-096794 28077355

[200] Andia I Martin JI Maffulli N. Advances with platelet rich plasma therapies for tendon regeneration. Expert Opin Biol Ther. (2018) 18:389–98. 10.1080/14712598.2018.1424626 29300106

[201] Hudgens JL Sugg KB Grekin JA Gumucio JP Bedi A Mendias CL. Platelet-rich plasma activates pro-inflammatory signaling pathways and induces oxidative stress in tendon fibroblasts. Am J Sports Med. (2016) 44:1931–40. 10.1177/0363546516637176 27400714 PMC4970921

[202] Bendinelli P Matteucci E Dogliotti G Corsi MM Banfi G Maroni P. Molecular basis of anti-inflammatory action of platelet-rich plasma on human chondrocytes: mechanisms of NF-κB inhibition *via* HGF. J Cell Physiol. (2010) 225:757–66. 10.1002/jcp.22274 20568106

[203] McNamee MJ Coveney CM Faulkner A Gabe J. Ethics, evidence based sports medicine, and the use of platelet rich plasma in the English premier league. Health Care Anal. (2018) 26:344–61. 10.1007/s10728-017-0345-7 28756518 PMC6208980

[204] Rachul C Rasko JEJ Caulfield T. Implicit hype? Representations of platelet rich plasma in the news media. PLoS ONE. (2017) 12:e0182496. 10.1371/journal.pone.0182496 28792974 PMC5549909

[205] Filardo G Kon E Di Matteo B Di Martino A Tesei G Pelotti P. Platelet-rich plasma injections for the treatment of refractory Achilles tendinopathy: results at 4 years. Blood Transfus. (2014) 12:533–40. 24960641 10.2450/2014.0289-13 PMC4212034

[206] Fitzpatrick J Bulsara M Zheng MH. The effectiveness of platelet-rich plasma in the treatment of tendinopathy: a meta-analysis of randomized controlled clinical trials. Am J Sports Med. (2017) 45:226–33. 10.1177/0363546516643716 27268111

[207] Pandey V Bandi A Madi S Agarwal L Acharya KK Maddukuri S. Does application of moderately concentrated platelet-rich plasma improve clinical and structural outcome after arthroscopic repair of medium-sized to large rotator cuff tear? A randomized controlled trial. J Shoulder Elbow Surg. (2016) 25:1312–22. 10.1016/j.jse.2016.01.036 27262412

[208] Slomski A. Platelet-rich plasma doesn't facilitate Achilles tendon healing. J Am Med Assoc. (2020) 323:701. 10.1001/jama.2020.1006 32096849

[209] Meyer U. The History of Tissue Engineering and Regenerative Medicine in Perspective. Fundamentals of Tissue Engineering and Regenerative Medicine. Berlin: Springer Berlin Heidelberg (2009).

[210] Law JX Liau LL Aminuddin BS Ruszymah BH. Tissue-engineered trachea: a review. Int J Pediatr Otorhinolaryngol. (2016) 91:55–63. 10.1016/j.ijporl.2016.10.012 27863642

[211] Chainani A Hippensteel KJ Kishan A Garrigues NW Ruch DS Guilak F. Multilayered electrospun scaffolds for tendon tissue engineering. Tissue Eng A. (2013) 19:2594–604. 10.1089/ten.tea.2013.0165 23808760 PMC3856877

[212] Van Der Merwe W Lind M Faunø P Van Egmond K Zaffagnini S Marcacci M. Xenograft for anterior cruciate ligament reconstruction was associated with high graft processing infection. J Exp Orthop. (2020) 7:79. 10.1186/s40634-020-00292-0 33026544 PMC7541808

[213] Dhammi IK. Rehan-Ul-Haq, Kumar S. Graft choices for anterior cruciate ligament reconstruction. Indian J Orthop. (2015) 49:127–8. 10.4103/0019-5413.152393 26015598 PMC4436475

[214] Chen T Jiang J Chen S. Status and headway of the clinical application of artificial ligaments. Asia Pac J Sports Med Arthrosc Rehabil Technol. (2015) 2:15–26. 10.1016/j.asmart.2014.11.001 29264235 PMC5730644

[215] Oryan A Moshiri A Meimandi-Parizi A. Graft selection in ACL reconstructive surgery. Curr Orthopaedic Pract. (2013) 24:321–33. 10.1097/BCO.0b013e31828b85cb

[216] Yin Z Chen X Chen JL Ouyang HW. Stem cells for tendon tissue engineering and regeneration. Expert Opin Biol Ther. (2010) 10:689–700. 10.1517/14712591003769824 20367125

[217] Aldana AA Abraham GA. Current advances in electrospun gelatin-based scaffolds for tissue engineering applications. Int J Pharm. (2017) 523:441–53. 10.1016/j.ijpharm.2016.09.044 27640245

[218] Lim J Razi ZRM Law JX Nawi AM Idrus RBH Chin TG. Mesenchymal stromal cells from the maternal segment of human umbilical cord is ideal for bone regeneration in allogenic setting. Tissue Eng Regen Med. (2017) 15:75–87. 10.1007/s13770-017-0086-6 30603536 PMC6171637

[219] Dai L Hu X Zhang X Zhu J Zhang J Fu X. Different tenogenic differentiation capacities of different mesenchymal stem cells in the presence of BMP-12. J Transl Med. (2015) 13:200. 10.1186/s12967-015-0560-7 26104414 PMC4479325

[220] Bhatia R Hare JM. Mesenchymal stem cells: future source for reparative medicine. Congest Heart Fail. (2005) 11:87–93. 10.1111/j.1527-5299.2005.03618.x 15860974

[221] Yuan T Zhang CQ Wang JH. Augmenting tendon and ligament repair with platelet-rich plasma (PRP). Muscles Ligaments Tendons J. (2013) 3:139–49. 24367773 PMC3838322

[222] Sevivas N Teixeira FG Portugal R Direito-Santos B Espregueira-Mendes J Oliveira FJ. Mesenchymal stem cell secretome improves tendon cell viability *in vitro* and tendon-bone healing *in vivo* when a tissue engineering strategy is used in a rat model of chronic massive rotator cuff tear. Am J Sports Med. (2018) 46:449–59. 10.1177/0363546517735850 29053925

[223] MacCarthy-Morrogh L Martin P. The hallmarks of cancer are also the hallmarks of wound healing. Sci Signal. (2020) 13:eaay8690. 10.1126/scisignal.aay8690 32900881

